# Anion exchange resins in phosphate form as versatile carriers for the reactions catalyzed by nucleoside phosphorylases

**DOI:** 10.3762/bjoc.16.212

**Published:** 2020-10-22

**Authors:** Julia N Artsemyeva, Ekaterina A Remeeva, Tatiana N Buravskaya, Irina D Konstantinova, Roman S Esipov, Anatoly I Miroshnikov, Natalia M Litvinko, Igor A Mikhailopulo

**Affiliations:** 1 Institute of Bioorganic Chemistry, National Academy of Sciences of Belarus, 220141 Minsk, Acad. Kuprevicha 5/2, Republic of Belarus; 2 Shemyakin and Ovchinnikov Institute of Bioorganic Chemistry, Russian Academy of Sciences, Miklukho-Maklaya 16/10, 117997 GSP-7, Moscow B-437, Russian Federation

**Keywords:** anion exchange resins, *N*^6^-benzyladenosine, cladribine, enzymatic glycosylation, kinetin riboside, nelarabine, α-ᴅ-pentofuranose-1-phosphates, phosphorolysis of nucleosides, purine nucleoside phosphorylases, recombinant *E. coli* uridine, thymidine

## Abstract

In the present work, we suggested anion exchange resins in the phosphate form as a source of phosphate, one of the substrates of the phosphorolysis of uridine, thymidine, and 1-(β-ᴅ-arabinofuranosyl)uracil (Ara-U) catalyzed by recombinant *E. coli* uridine (UP) and thymidine (TP) phosphorylases. α-ᴅ-Pentofuranose-1-phosphates (PF-1Pis) obtained by phosphorolysis were used in the enzymatic synthesis of nucleosides. It was found that phosphorolysis of uridine, thymidine, and Ara-U in the presence of Dowex^®^ 1X8 (phosphate; Dowex-*n*Pi) proceeded smoothly in the presence of magnesium cations in water at 20–50 °C for 54–96 h giving rise to quantitative formation of the corresponding pyrimidine bases and PF-1Pis. The resulting PF-1Pis can be used in three routes: (1) preparation of barium salts of PF-1Pis, (2) synthesis of nucleosides by reacting the crude PF-1Pi with an heterocyclic base, and (3) synthesis of nucleosides by reacting the ionically bound PF-1Pi to the resin with an heterocyclic base. These three approaches were tested in the synthesis of nelarabine, kinetin riboside, and cladribine with good to excellent yields (52–93%).

## Introduction

Diverse variants of enzymatic syntheses of nucleosides using nucleoside phosphorylases as biocatalysts have been repeatedly described in original studies and discussed in a number of recent reviews (see, e.g., [1–15]). In the late forties and early fifties of the last century, α-ᴅ-pentofuranose-1-phosphates (PF-1Pis) were isolated from the phosphorolysis of natural nucleosides catalyzed by nucleoside phosphorylases and their structures and enzymatic reactions with heterocyclic bases were studied in detail [16–20]. The transglycosylation reaction, that is the transfer of a carbohydrate fragment from a nucleoside donor to another heterocyclic base acceptor, was discovered by Kalckar in 1945 [16] (for reviews of pioneering works, see [17–18]). In a brief letter to the editor Kalckar reported for the first time that specific inosine nucleosidase from rat liver catalyzes the reversible reaction “inosine + phosphate ⇌ hypoxanthine + ribose-1-phosphate”: the equilibrium of this reaction in water at pH 6.5 is displaced to the nucleoside synthesis. Moreover, he made the very interesting observation that the reaction of hypoxanthine and ribose-1-phosphate reached equilibrium after 20 min at a 70% concentration of inosine and “The synthesis of inosine would probably proceed further if all traces of inorganic phosphate were removed, since with equimolar amounts of inosine and phosphate as starting material it was found that only 10 per cent of the riboside undergoes phosphorolysis at pH 6.5” [16].

Presently, the observation made by Kalckar is one of the hot issues from a viewpoint of the development of efficient enzymatic synthesis of nucleosides employing nucleoside phosphorylases as biocatalysts. The enzymatic reaction of PF-1Pis and heterocyclic bases is a key step of the transglycosylation strategy for the synthesis of biologically important nucleosides. The reactions of phosphorolysis and interaction of the resulting PF-1Pis with heterocyclic bases are reversible, and a thorough optimization of both transformations is necessary in most cases, especially upon the implementation of both transformations in a one-pot mode, in order to obtain the desired nucleoside in high or acceptable yield (see, e.g., recent papers [21–22] and references cited therein).

The classical two-stage version of the enzymatic transglycosylation reaction [16–20], as well as one-pot synthesis, and the more sophisticated option employing two cross-glycosylation transformations for nucleoside synthesis [23–27], seemed less attractive and more complicated in comparison with the enzymatic reaction of a heterocyclic base with an individual PF-1Pi [17–18,27]. However, it is known that most of purine heterocycles are poorly soluble in aqueous phosphate buffers, and the use of a cross-glycosylation scheme, i.e., a purine nucleoside as a donor of the purine base and another nucleoside as a source of a PF-1Pi, could solve this problem in some cases, despite a more complex optimization process. On the whole, the choice of the methodology for the production of biologically important nucleosides depends on a number of factors and the problems associated with the reversibility of enzymatic phosphorolysis/synthesis reactions, along with the solubility of heterocyclic bases and accessibility of PF-1Pis. Despite all this, in a number of cases these problems have been successfully solved and, as a result, the desired base and/or sugar-modified nucleosides were obtained in good yields.

In spite of the obvious progress in using the transglycosylation reaction for the synthesis of biologically important nucleosides, the idea of researching and developing an effective method for the enzymatic or chemoenzymatic synthesis of PF-1Pis has again attracted the attention of researchers. Recently, the phosphorolysis of commercially available uridine (Urd), thymidine (Thd), and 1-(β-ᴅ-arabinofuranosyl)uracil (Ara-U) under the action of recombinant (i) uridine phosphorylase (UP) and thymidine phosphorylase (TP) from *E. coli* [28], and (ii) thermostable pyrimidine nucleoside phosphorylase (PyNP-Y04 and PyNP-Y02) [29] was studied and the corresponding Rib-1Pi, dRib-1Pi, and Ara-1Pi were obtained in the form of barium salts in yields within 13–37%. At the same time, the strategy by Hennen and Wong [30–31] was used to synthesize Rib-1Pi and dRib-1Pi consisting in the chemical synthesis of *N*^7^-methyl derivatives of guanosine (*N*^7^Me-Gua) [30,32–35] and 2'-deoxyguanosine (*N*^7^Me-dGua) [31,34,36–37], and the desired Rib-1Pi and dRib-1Pi were obtained in high yields [35–37]. The final aim of these studies was the synthesis of biologically important purine and pyrimidine nucleosides. A scrutiny of the results of these studies vs the transglycosylation reaction showed that both methods have certain advantages along with obvious disadvantages, namely, problems with obtaining individual PF-1Pis (in particular removal of excess inorganic phosphate [28–29]) and moderate or low yields, on the one hand, and the need to obtain *N*^7^Me-Gua [30,32–35] and *N*^7^Me-dGua [31,34,36–38] by chemical methylation, the failure of Ara-1P synthesis [34], and the need to utilize *N*^7^-methylguanine, on the other.

In a recently published work by Serianni et al. [39] a multienzyme one-pot methodology was investigated in detail and applied for the synthesis of Rib-1Pi and dRib-1Pi. This methodology consisted in the phosphorolysis of inosine (Ino) or 2′-deoxyinosine (dIno) by purine nucleoside phosphorylase (PNP) accompanied by the xanthine oxidase-catalyzed oxidation of the hypoxanthine formed to uric acid [40] and H_2_O_2_, and the latter was converted to H_2_O and oxygen by catalase preventing thereby the reverse formation of the starting Ino. The Rib-1Pi (2Na^+^) was isolated from the reaction mixture in 74% yield (32 IU of PNP per 1 mmol of Ino; 50 mM Na-phosphate buffer, pH 7.4; 37 °C, 48 h; conversion >95%) and employed for the synthesis of adenosine (Ado; PNP, water, 3 days at 37 °C; conversion 95%), guanosine (Guo; PNP, water 90% and DMSO 10%, 9 days at 37 °C; conversion 40%), and uridine (Urd; UP, water, 20 h at 37 °C; conversion 85%). In the synthesis of dRib-1Pi (2Na^+^) (80 IU of PNP per 1 mmol of dIno; 50 mM Na-phosphate buffer, pH 7.4; 22 °C; 20%), which was tested in the synthesis of dAdo (conversion 60%), dGuo (5%), Urd (52%), and thymidine (Thd; 60%), a number of unexpected transformations was observed. The most puzzling was the PNP-mediated isomerization of dIno into 7-(2-deoxy-β-ᴅ-ribofuranosyl)hypoxanthine (2'd-*N*^7^-inosine) through reversal condensation of dRib-1P and hypoxanthine. The formation of dRib-1P and 2'd-*N*^7^-inosine in yields of 29% and 49%, respectively, was estimated in the reaction mixture starting from dIno (100 IU of PNP per 1 mmol of dIno) in 10 mM Na-phosphate buffer (pH 7.4) at 22 °C for 6 h. Noteworthy, the formation of the 2'd-*N*^7^- and 2'd-*N*^9^-glycosides of *N*^2^-acetylguanine (^Ac^Gua) was observed in the transglycosylation of ^Ac^Gua using dGuo/*E. coli* PNP (67.5 IU per 1 mmol base) for an in situ generation of dRib-1Pi (5 mM K-phosphate buffer, pH 7.4; 45 °C) [41]. It was shown that the *N*^7^-isomer formed predominantly at the beginning of the reaction and the ratio of *N*^7^- and *N*^9^-isomers displaced gradually to the latter getting equilibrium at ca. 1:4.8 ratio after 24 h.

## Results and Discussion

**Scheme 1:**
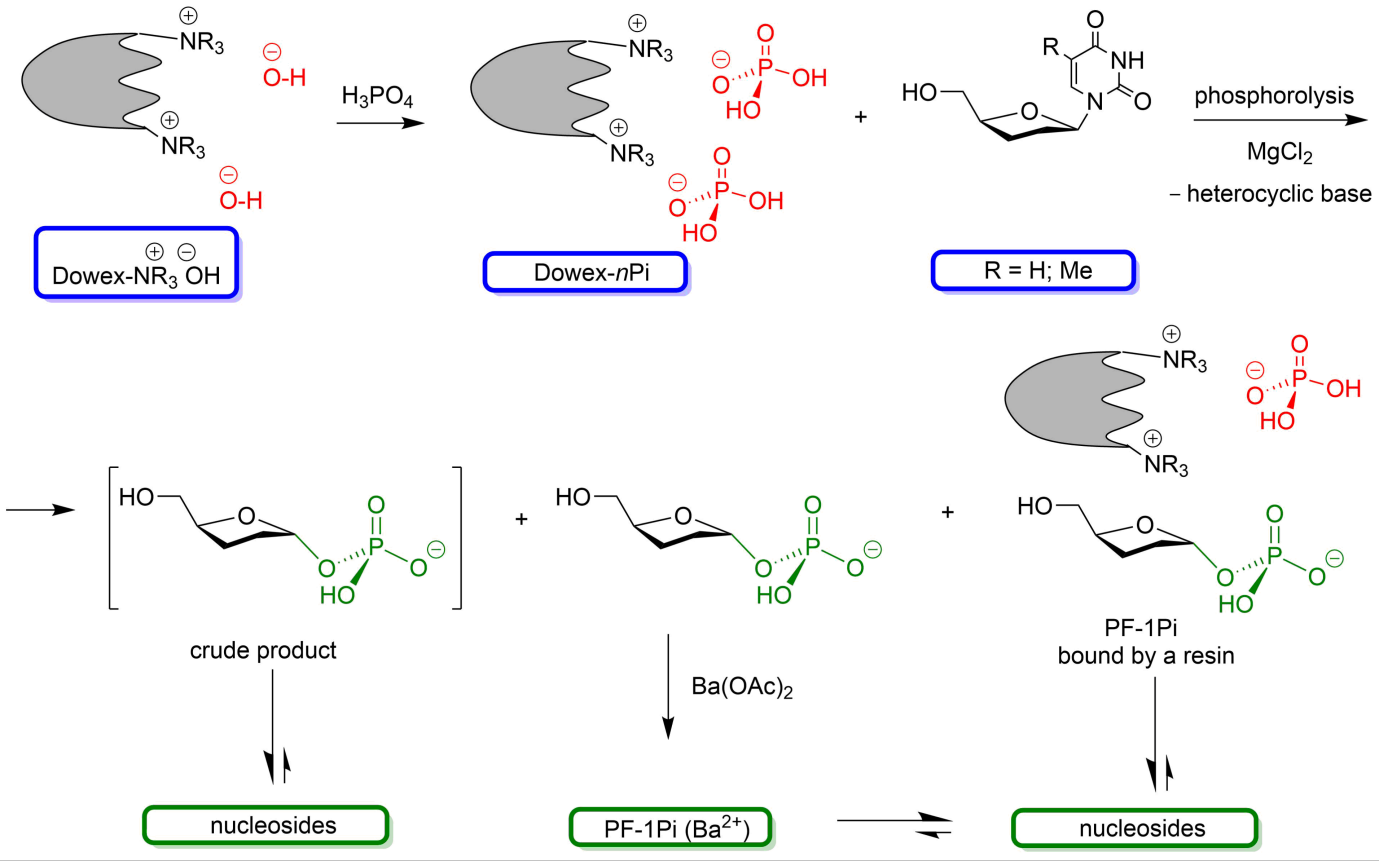
General scheme of the suggested synthesis of nucleosides employing the enzymatic phosphorolysis of pyrimidine nucleosides by recombinant *E. coli* uridine (UP) and thymidine (TP) phosphorylases to prepare the relevant α-ᴅ-pentofuranose-1-phosphates (PF-1Pis) and the use of them as either crude product, barium salt, or ionically bound by the resin in the enzymatic syntheses of nucleosides.

The analysis of the experimental works discussed above prompted us to start the search for simple and at the same time effective methods for the enzymatic synthesis of α-ᴅ-pentofuanose-1-phosphates (PF-1Pis) as key intermediates for the synthesis of nucleosides according to Kalсkar by phosphorolysis of readily available pyrimidine and purine nucleosides. With this aim in view, studies have initiated on the synthesis of Rib-1Pi, dRib-1Pi, and Ara-1Pi based on the corresponding pyrimidine nucleosides and *E. coli* UP and TP in the presence of Dowex^®^ 1X8 in phosphate form (100–200 mesh; Dowex-*n*Pi) as a source of inorganic phosphate in water instead of phosphate buffer. The initial task was to identify the conditions for the quantitative phosphorolysis of the starting nucleosides to obtain PF-1Pis, on the one hand, and to compare their use as crude product, as barium salt, or in the form of ionically bound form with the anion exchange resin in the enzymatic syntheses of biologically important purine nucleosides, on the other hand (Scheme 1).

As might be expected, the replacement of phosphate buffer with Dowex-*n*Pi in the phosphorolysis of uridine (Urd), thymidine (Thd), and 1-(β-ᴅ-arabinofuranosyl)uracil (Ara-U) slowed down the reaction rate and increased the time required for the complete breakdown of the glycoside bond of the starting nucleoside. In order to find optimal conditions for the phosphorolysis reaction in the presence of Dowex-*n*Pi, we analyzed some of the results of previous studies in terms of the role of the amount of bioсatalyst, substrate activity of heterocyclic bases, and a number of other factors on the efficiency of the enzymatic synthesis of nucleosides.

### The role of diverse factors in the nucleoside synthesis catalyzed by nucleoside phosphorylases

The results of our previous studies and observations also motivated our search for new variants of nucleoside synthesis using nucleoside phosphorylases, since they indicated a complex dependence of the efficiency of enzyme-mediated phosphorolysis and nucleoside synthesis on a number of different factors (see [41–42], and the works cited therein). Thus, e.g., in our work on the synthesis of purine arabinosides by condensation of chemically prepared Ara-1Pi (Li^1+^) with 2-fluoroadenine, hypoxanthine, and *O*^6^-methylguanine (OMG) catalyzed by *E. coli* PNP under comparable conditions (water solution, 45–55 °C), a significant difference was found in the preparation efficiency of the corresponding nucleosides depending on the structure of the heterocyclic base [42]. The following results regarding the base ⇌ nucleoside equilibrium point (percent of nucleoside in the reaction mixture and the time required to establish an equilibrium) of the reaction and the preparation of the individual products (≥98%) were obtained: Fludarabine 94% after 3 h, yield 77%; hypoxanthine-arabinoside 90% after 168 h, yield 81%, and nelarabine 50% after 36 h, yield 40%, respectively [42]. Earlier, Krenitsky et al. described the synthesis of nelarabine [43] and fludarabine [44] using the transglycosylation reaction of OMG (Ara-U as a donor of Ara-1Pi) and 2-fluoroadenine [1-(β-ᴅ-arabinofuranosyl)cytosine (Ara-C) + cytidine deaminase for an in situ generation of Ara-1Pi], respectively, and the recombinant *E. coli* UP and PNP as biocatalysts in an one-pot mode. Unexpectedly, a significantly lower efficiency of the transarabinosylation of 2-fluoroadenine (UP 8.7 IU and PNP 93.3 IU; 4 mM K-phosphate buffer, pH 7.0; 864 h at 35 °C; 14%) [44] vs the condensation of Ara-1Pi and 2-fluoroadenine (water, 360 IU of PNP; 3 h at 55 °C; 77%) [42], and a comparable efficiency of nelarabine synthesis, viz., PNP 37,800 IU; 10 mM K-phosphate buffer, pH 7.4; 18 h at 60 °C; 53% [43] vs PNP 49 IU, water, 36 h at 45 °C; 40% [42] (see Supporting Information File 1 for combined data on the enzymatic synthesis of nucleosides, SI-6). The reaction conditions under comparison are rather different, but, nonetheless, dramatic differences in the PNP quantity (all data are given in IU per base) in the case of nelarabine syntheses did not allow us a direct comparison.

**Figure 1:**
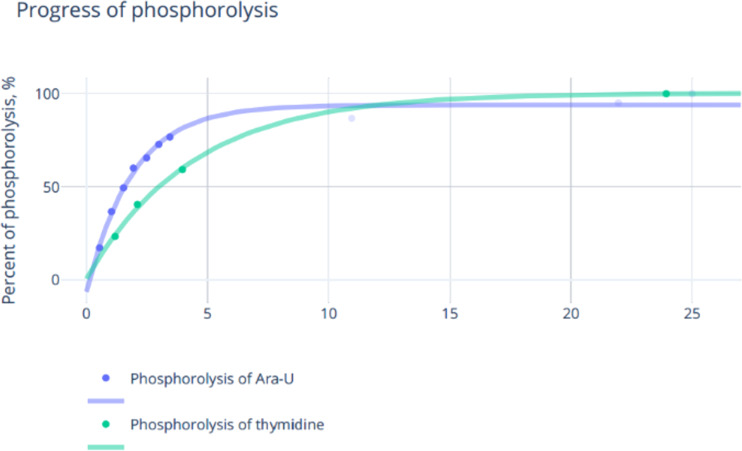
Phosphorolysis (5.0 mM K-phosphate buffer, pH 7.0; 23 °C) of Ara-U and thymidine (Thd) catalyzed by the corresponding *E. coli* enzymes.

These hardly consonant data prompted us to test once more the synthesis of nelarabine and related nucleosides using transglycosylation reactions. First of all, the phosphorolysis of Ara-U and thymidine in 5 mM K-phosphate buffer (pH 7–8) at room temperature was tested at different quantities of UP and TP, respectively, and it was found that an almost complete phosphorolysis ended after 10–25 h employing about 1,000 IU of the enzyme per 1 mmol of substrate (Figure 1). Apparently, a combination of such factors as the molarity of the phosphate buffer and the amount of enzymes per 1 mmol of substrate allows the phosphorolysis reactions to proceed by almost entirely.

**Scheme 2:**
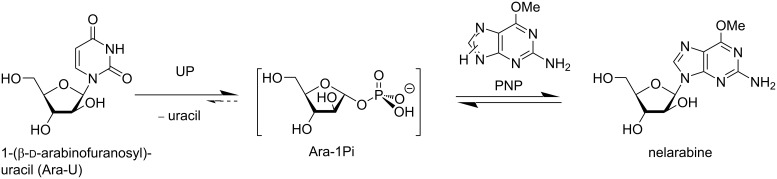
Transarabinosylation of *O*^6^-methylguanine (OMG) employing Ara-U as a donor of the Ara-1Pi (1:1.5 molar ratio of OMG and Ara-U) and the recombinant *E. coli* UP (5,000 IU per 1 mmol of Ara-U) and PNP (10,000 IU per 1 mmol of base) as biocatalysts at 23 °C for 50 h.

Taking into account the quantity of UP and PNP in the synthesis of nelarabine employed by Krenitsky et al. [43] as well as the somewhat lower catalytic efficiency of *E. coli* PNP vs that of *E. coli* UP for the relevant substrates, we tested the similar transglycosylation reaction using 5,000 IU of UP per 1 mmol of Ara-U and 10,000 IU of PNP per 1 mmol of *O*^6^-methylguanine (OMG) at a 1.5:1.0 molar ratio of substrates in 5.0 mM K-phosphate buffer (pH 8.0) at room temperature. Under these reaction conditions, the Ara-U phosphorolysis was complete in ca. 1 h, while the next stage, viz., the reaction of the formed Ara-1Pi and the base proceeded very quickly, reaching a maximum after 50 min at сa. 45% nucleoside concentration then began to decrease to 36%, and after 50 h equilibrium was established at 34% of nucleoside. Notably, a reverse synthesis of Ara-U was not observed (Scheme 2). These results indicate that the transarabinosylation reaction, as in the case of Krenitsky et al. [43] as well as described by us earlier [42], is difficult to manage and careful optimization is necessary in order to achieve a higher yield of nelarabine. It is noteworthy that an application of such high concentrations of UP did not shift the equilibrium to the product formation.

**Figure 2:**
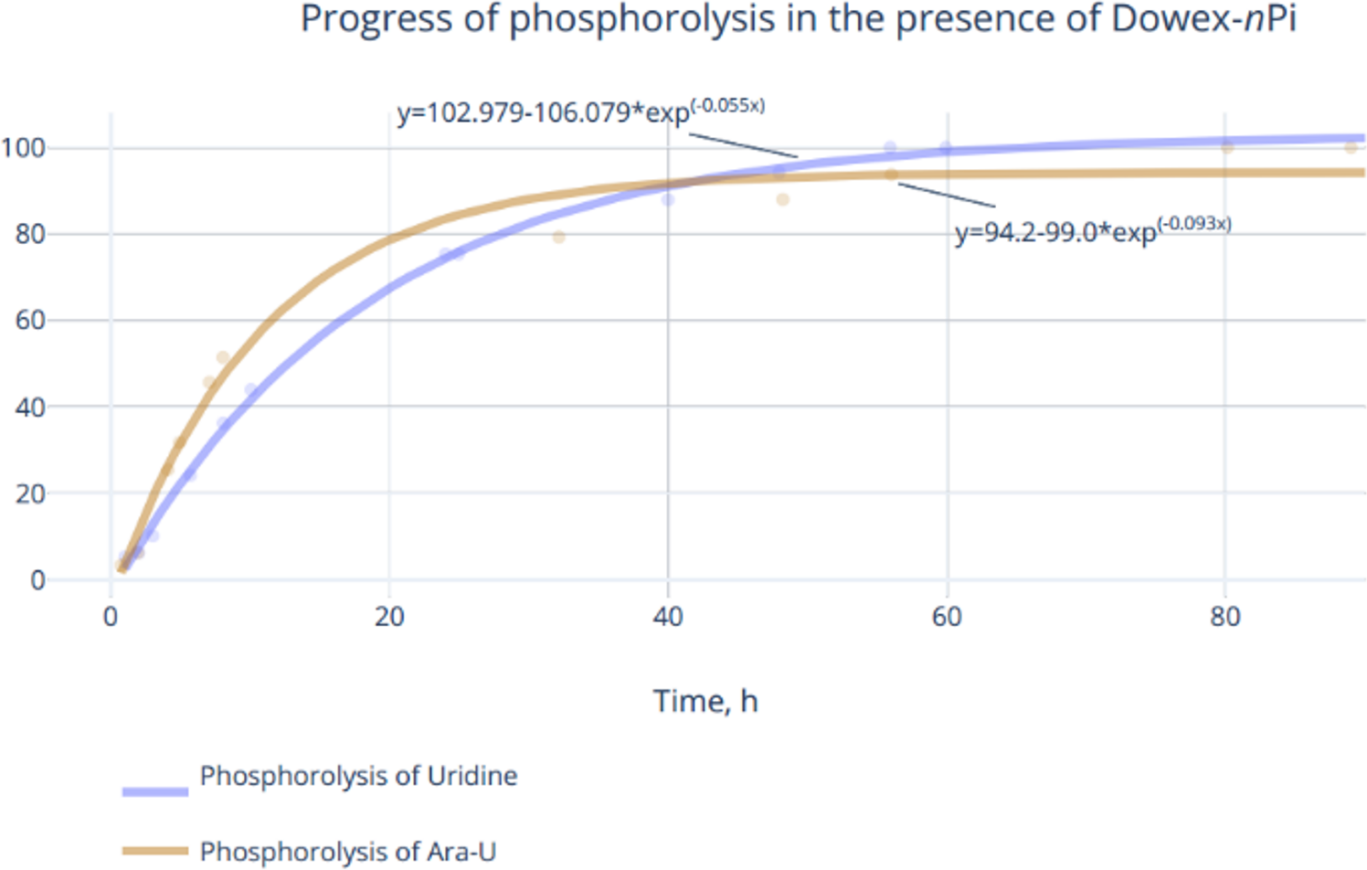
Optimized conditions of phosphorolysis of Ara-U: 0.20 mmol of Ara-U in distilled water (30 mL) containing Dowex 1X8 (phosphate; 7 mL of wet resin), MgCl_2_·6H_2_O, i.e., 0.20 mmol of MgCl_2_ per 1 mmol of Ara-U), and *E. coli* UP (105 IU). Urd: 0.16 mmol of Urd in distilled water (30 mL) containing Dowex 1X8 (phosphate; 5 mL of wet resin), MgCl_2_·6H_2_O, i.e., 0.16 mmol of MgCl_2_ per 1 mmol of Urd) and *E. coli* UP (100 IU).

The replacement of the 5 mM K-phosphate buffer in this reaction with Dowex-*n*Pi resulted in slowing down of the Ara-U phosphorolysis and as a consequence nelarabine formation, and an increase in the amount of UP did not lead again to the proportional increase in nelarabine synthesis. On the contrary, the addition of magnesium ions to the reaction mixture (0.1–0.5 mol of Mg^2+^ per 1 mol of nucleoside) made it possible to enhance the rate of the phosphorolysis and, at the same time, to achieve practically quantitative phosphorolysis of Ara-U and uridine to respective Ara-1Pi and Rib-1Pi in 54–72 h (conversion ≥95%) (Figure 2). Noteworthy, the enhancing effect of magnesium ions on metabolic transformations of nucleosides [45], in particular on the phosphorylation of the primary hydroxy group of pentoses, catalyzed by ribokinases [46], as well as on the cascade synthesis of nucleosides starting from pentoses [47–50] has been described. Based on the results of these experiments a simple preparative method for the synthesis of Rib-1Pi and Ara-1Pi in form of barium salts has been developed (vide infra).

#### The use of PF-1Pi as (i) crude product of nucleoside phosphorolysis in the presence of anion exchange resin in phosphate form and (ii) as barium salt

The resulting PF-1Pis have been studied in three ways: (1) after completion of the phosphorolysis, Dowex-*n*Pi was filtered off, the recombinant *E. coli* PNP and a purine base were added to the filtrate, and the formation of the desired purine nucleoside was monitored by TLC and HPLC, (2) the resulting PF-1Pis were isolated as barium salts from the supernatant remaining after removal of Dowex-*n*Pi in ca. 90% yield and studied in the synthesis of nucleosides, and (3) a part of PF-1Pi bonded ionically to an anion exchange resin was tested as a source of PF-1Pi in the enzymatic synthesis of a desired nucleoside.

The possibility of using the filtrate obtained in the 1st way as a source of PF-1Pis was tested by the enzymatic synthesis of nelarabinе and kinetin riboside (KR). The choice of these two nucleosides was primarily determined by their biological activities: nelarabine was approved by the Food and Drug Administration (FDA, USA) in 2005 as an antileukemia drug [51–56]. KR belonging to a family of phytohormones [57], displays a broad spectrum of biological activity [58–60], and in the form of pro-tides revealed an interesting spectrum of activity against neurodegenerative diseases [60]. The reaction of the enzymatic transglycosylation was used by Krenitsky et al. in the practical synthesis of nelarabine (vide supra), whereas the preparation of KR has been reported earlier (i) by employing the selected whole *E. coli* cells [61–62] and briefly described (ii) by using the recombinant *E. coli* nucleoside phosphorylases [41] as biocatalysts, respectively.

#### Synthesis of nelarabine

**Scheme 3:**
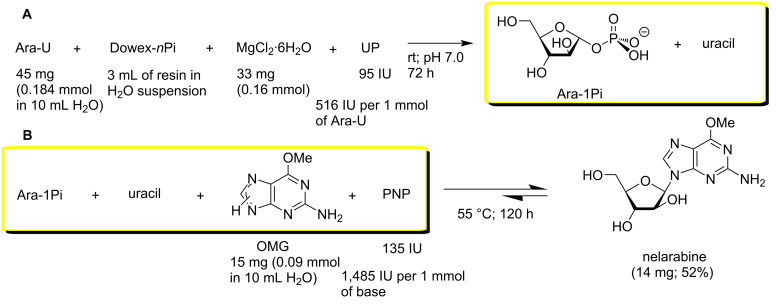
Synthesis of nelarabine with intermediate preparation of crude Ara-1Pi.

The reaction of *O*^6^-methylguanine (OMG) with the crude Ara-1Pi remaining in the reaction mixture of the Ara-U phosphorolysis after removing of Dowex-*n*Pi (Scheme 3) and with purified Ara-1Pi (Ba^2+^) was studied in the synthesis of nelarabine. The phosphorolysis of Ara-U in the presence of Dowex-*n*Pi catalyzed by *E. coli* UP at room temperature for 72 h resulted in the formation of uracil in a yield of ca. 97% (Scheme 3A). The Dowex-*n*Pi resin was filtered off, washed with small quantity of distilled water, OMG and *E. coli* PNP were added to the combined filtrate and washings, and the resulting new reaction mixture was stirred at 55 °C for 120 h (Scheme 3B); the progress of the reaction was monitored by TLC and HPLC. The ca. 1:2 (mol) ratio of the purine base to the crude Ara-1Pi was chosen from the calculation of complete phosphorolysis assuming that the resulting Ara-1Pi remains (ca. 90%; vide infra) in aqueous solution and not ionically bound (absorbed) by the Dowex resin. Under these reaction conditions, the conversion of the purine base to nelarabine was ca. 60%, and after conventional work-up and silica gel column chromatography the desired nucleoside was obtained in 52% yield. The rather long reaction time of the phosphorolysis and the Ara-1P-base coupling is mainly connected with the low quantity of the employed UP and PNP in both transformations. Indeed, it is noteworthy that in the work of Krenitsky et al. [43] in the transarabinosylation reaction the ratio of substrates to the relevant enzymes (throughout in IU per 1 mmol of substrate) was UP 1,890 IU for Ara-U and PNP 37,780 IU for the heterocyclic base, whereas in our experiment the corresponding values were 516 IU and 1,485 IU for UP and PNP, respectively (Scheme 3). Taken into account that the yields of nelarabine in both cases were rather similar, our approach appears to be more attractive.

In the framework of the 2nd way, the Ara-1Pi (Ba^2+^) was obtained from the phosphorolysis of Ara-U (170 mg, 0.7 mmol) in the presence of Dowex-*n*Pi (6 mL; 1.2 mequiv/mL) and MgCl_2_·6H_2_O (0.35 mmol) catalyzed by UP (105 IU; 150 units per 1 mmol of substrate) at 40 °C for 72 h giving rise to the formation of uracil in practically quantitative yield (>97%) according to HPLC analysis. The reaction mixture was worked up as described above, the filtrate was kept in a refrigerator at 4 °C overnight, the precipitate formed was filtered off and the filtrate was mixed with water/ammonia solution of Ba(OAc)_2_ that was stored at 4 °C overnight. The precipitate formed was centrifuged off, to the supernatant 5 volumes of ethanol added, and the mixture kept overnight in a refrigerator. The precipitate formed was filtered off, washed with ethanol, dried in vacuum under CaCl_2_ at room temperature to give Ara-1Pi (Ba^2+^) as white powder (244 mg, 96%; TLC: iPrOH/25% NH_4_OH/water, 11:2:5 (vol), *R*_f_ 0.30, development by heating). The NMR data were similar to those published earlier [29].

The barium salt of Ara-1Pi (73 mg, 0.2 mmol) and *E. coli* PNP (280 IU; 2,333 IU per 1 mmol of heterocyclic base) were added to an aqueous solution of OMG (20 mg, 0.12 mmol, dissolved by heating) in distilled water (50 mL) at room temperature, and the reaction mixture was gently stirred at 47 °C for 48 h, with monitoring the product formation by HPLC. After 48 h, the conversion of the base into nucleoside reached equilibrium at 85% of the latter. The turbid (not reacted starting base according to TLC and barium phosphate) reaction mixture was filtered, the filtrate evaporated, and the residue treated with EtOH (20 mL). The non-dissolved fine powder was filtered off and the filtrate evaporated and co-evaporated with EtOH (2 × 20 mL). The obtained residue was dissolved in MeOH (10 mL), mixed with silica gel (2 mL), evaporated and the residue was put on the top of a silica gel column (1.5 × 23 cm) prepared in EtOAc. The fractions containing the nucleoside were combined, evaporated and dried to afford the powdered product (25 mg; 66%) of 95.5% purity (HPLC), that was crystallized from MeCN to give nelarabine (19 mg; 53%; 99.0% purity by HPLC). For NMR data, see Supporting Information File 1, SI-1, SI-2 and SI-3).

#### Synthesis of kinetin riboside (KR)

**Scheme 4:**
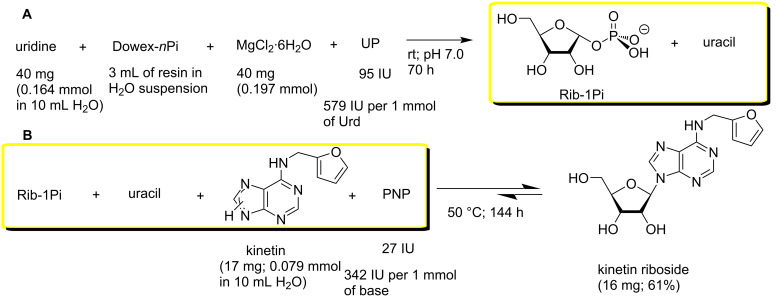
Synthesis of kinetin riboside with intermediate preparation of crude Rib-1Pi.

The synthesis of KR was performed essentially as described above for nelarabine. The first step of the synthesis, i.e., phosphorolysis of uridine catalyzed by *E. coli* UP, was realized in water milieu in the presence of Dowex-*n*Pi and MgCl_2_ at room temperature for 72 h to afford Rib-1Pi practically in quantitative yield (uridine → uracil conversion >96%) (Scheme 4). The second step of the synthesis, viz., condensation of the formed crude Rib-1Pi with kinetin catalyzed by PNP proceeded very slowly under the employed conditions attaining the formation of KR in 72% yield after incubation of the reaction mixture at 50 °C for 144 h. The very low substrate activity of kinetin towards *E. coli* PNP compared with the related *N*^6^-benzyladenine (vide infra) was one of the reasons for this observation. Conventional work-up of the reaction mixture followed by silica gel column chromatography gave KR in 61% yield of 98.0% purity according to HPLC.

We have earlier noticed that the enzyme-mediated reaction of analogs of natural purine bases with PF-1Pis (Ba^2+^) proceeded much faster and resulted in a high conversion yield in Tris·HCl buffer compared with the same reaction in water [28] (cf. [18–19]). The preparation of Rib-1Pi (Ba^2+^) was performed by analogy with the synthesis of Ara-1Pi (Ba^2+^) to give the white powder in 96% yield. The ^1^H and ^13^C NMR spectra of the product were analogous with those published earlier [28]. The reaction of Rib-1Pi (Ba^2+^) with kinetin (15 mg, 0.07 mmol) in 50 mM Tris·HCl buffer, at pH 8.0 water solution (40 mL) in the presence of the *E. coli* PNP (264 IU per 1 mmol of base) at 47 °C for 48 h resulted in the formation of KR in ca. 97% conversion and an isolated yield of 93% yield (crystallized from MeCN; 99.0% purity according to HPLC; for NMR data, see Supporting Information File 1, SI-1, SI-2 and SI-4).

#### The capacity of the anion exchange resins to bind PF-1Pi

As noted above, when studying Ara-U phosphorolysis catalyzed by *E. coli* UP in the presence of Dowex-*n*Pi, we found that (1) about 10% of the resulting Ara-1Pi was bound ionically and adsorbed by the resin and (2) the addition of the resin containing bound Ara-1Pi to an aqueous solution of *O*^6^-methylguanine (OMG) with *E. coli* PNP gave rise to the formation of nelarabine. This observation indicated the possibility of using the anion exchange resin loaded with ionically bound Ara-1Pi as shuttle of a key substrate for the enzymatic synthesis of nucleosides catalyzed by nucleoside phosphorylases. It is obvious that having succeeded in implementing such a scheme for the synthesis of nucleosides depends on the ability of the anion exchange resin to bind PF-1Pis formed by phosphorolysis. With this aim in view, we conducted preliminary experiments to assess QAE Sephadex A-25, BIO-RAD AG 1-X2 (200–325 mesh), and DEAE-cellulose (100–200 mesh) for their ability to bind PF-1Pis vs that of Dowex-*n*Pi.

A standard experiment included phosphorolysis of uridine by *E. coli* UP in the presence of the resins in phosphate form, and after completion of the reaction (conversion was more than 90%), the resin was filtered off, washed with distilled water, and added to a solution of *N*^6^-benzylaminopurine (BAP) in water also containing *E. coli* PNP. The formation of *N*^6^-benzylaminopurine riboside (BAPR; *N*^6^-benzyladenosine) was monitored by HPLC. A reference BAPR sample was obtained in 67% yield by the transglycosylation of BAP using a combination of guanosine and *E. coli* PNP to generate in situ Rib-1Pi (for the synthesis and NMR data, see Supporting Information File 1, SI-1, SI-2 and SI-5). It should be noted that (i) in contrast to the synthesis of nelarabine, an equilibrium BAP ⇌ BAPR is displaced to the nucleoside formation in the employed reaction conditions, and (ii) the cytokinine nucleoside BAPR shows a wide range of biological activities, which attracted our interest in this compound [57]. It was found that in the presence of QAE Sephadex A-25, BAPR formation reached 67%, while in the case of BIO-RAD AG 1-X2 and DEAE-Cellulose, the yield was 25 and 23%, respectively. Quite unexpectedly in the experiment with Dowex-*n*Pi the conversion of the base into BAPR was only 3% (all data from HPLC analysis) (Table 1).

**Table 1:** Synthesis of *N*^6^-benzyladenosine (BAPR) employing Rib-1Pi ionically bound with indicated resins (phosphate form; after attaining more that 90% uridine phosphorolysis by *E. coli* UP at rt and isolation from the reaction mixture) and *N*^6^-benzylaminopurine (BAP) catalyzed by *E. coli* PNP under standard reaction conditions.^a^

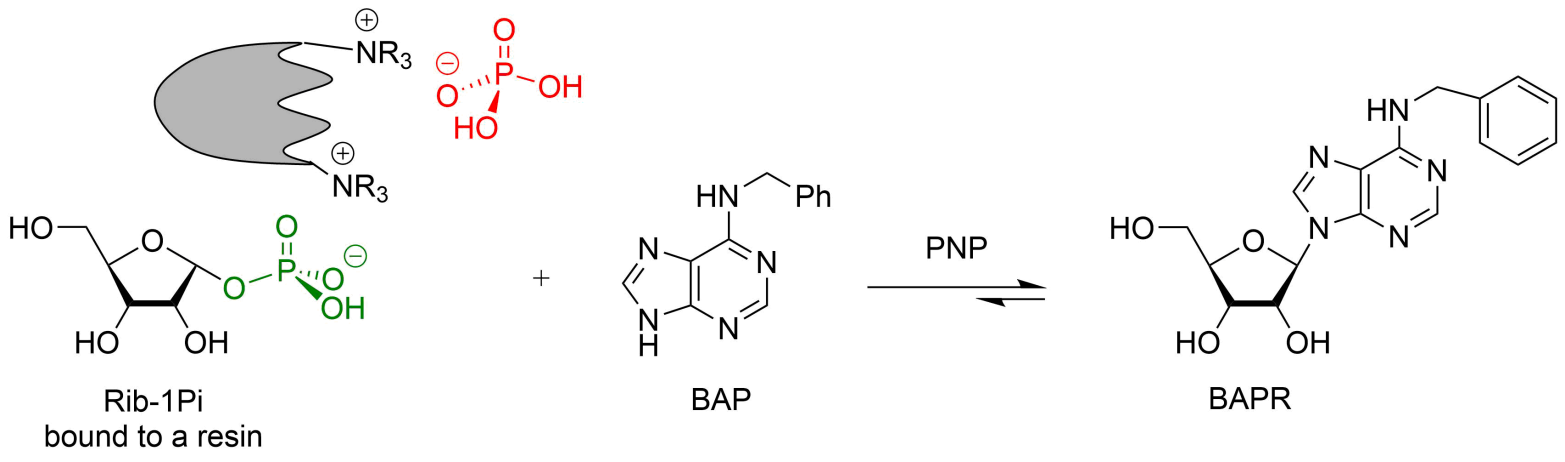	

Type of anion exchange resin (conversion of BAP → BAPR; % )	HPLC picture in standard reaction conditions

DEAE cellulose (23%; *t*_R_ 7.17 and 9.19 min)	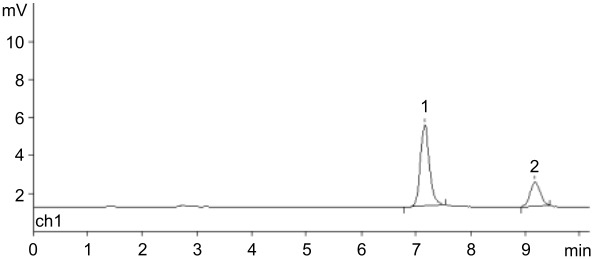
BIO-RAD AG 1-X2 (25%; *t*_R_ 7.16 and 9.18 min)	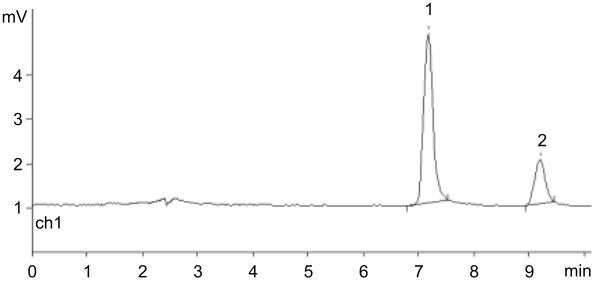
QAE Sephadex A-25 (67%; *t*_R_ 7.18 and 9.20 min)	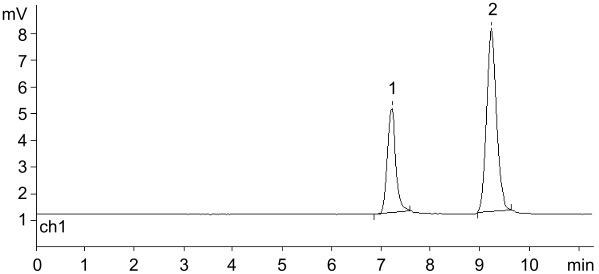
Dowex® 1x8 (ca. 3%; *t*_R_ 7.40 and 9.40 min)	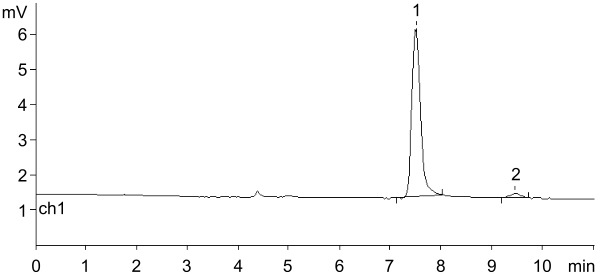

^a^Standard conditions: water; rt; percent of conversion according to HPLC: 25% MeCN in H_2_O, λ = 260 nm, 0.7 mL/min; *t*_R_ = 7.19 and 9.1 min for the base and nucleoside, respectively.

The results show that, depending on the structure of the matrix, phosphate anion exchange resins can be used as a source of inorganic phosphate ion in the reaction of enzymatic phosphorolysis of nucleosides (e.g., Dowex-*n*Pi) or to perform a dual function, viz., supply the phosphate ion in the phosphorolysis reaction and simultaneously accept the resulting PF-1Pi (e.g., QAE Sephadex A-25-*n*Pi) and liberate it as a key substrate in the enzymatic synthesis of nucleosides.

#### The use of dRib-1Pi (Ba^2+^) and dRib-1Pi bound to the QAE Sephadex A-25 in cladribine synthesis

The dual function of QAE Sephadex A-25 noted above was tested in the enzymatic synthesis of cladribine using dRib-1Pi as a barium salt and bound to the resin. Chemical synthesis of this nucleoside, which has a wide spectrum of pharmacological activities [6,63–65], is wearisome [5,66]. This evidence prompted us to engage in the design and development of an efficient biotechnological synthetic route.

Cladribine has been known for over 30 years as antineoplastic (Leustatine^®^) and anti-multiple sclerosis agent (for a few leading references, see [67–70]). The compound was approved by the FDA (USA) in 2006 as a drug against various types of leukemia, and recently, in 2019, it was approved (MAVENCLAD^®^) after intensive clinical testing for the treatment of relapsing forms of multiple sclerosis in adults. Its enzymatic synthesis using mainly nucleoside phosphorylases has been described in a number of publications (see, e.g., [25,37,71–75]) and the results of the present study are combined in Table 2.

**Table 2:** Condensation of ^2Cl^Ade with dRib-1Pi (Ba^2+^) and with dRib-1Pi ionically bound by QAE Sephadex A-25-*n*Pi catalyzed by *E. coli* PNP (water; at 40–50 °C; HPLC: column Agillent Eclipse Plus C18 (4.6 × 150 mm, 5 μm), 16% MeCN in H_2_O, λ = 260 nm, 0.7 mL/min).

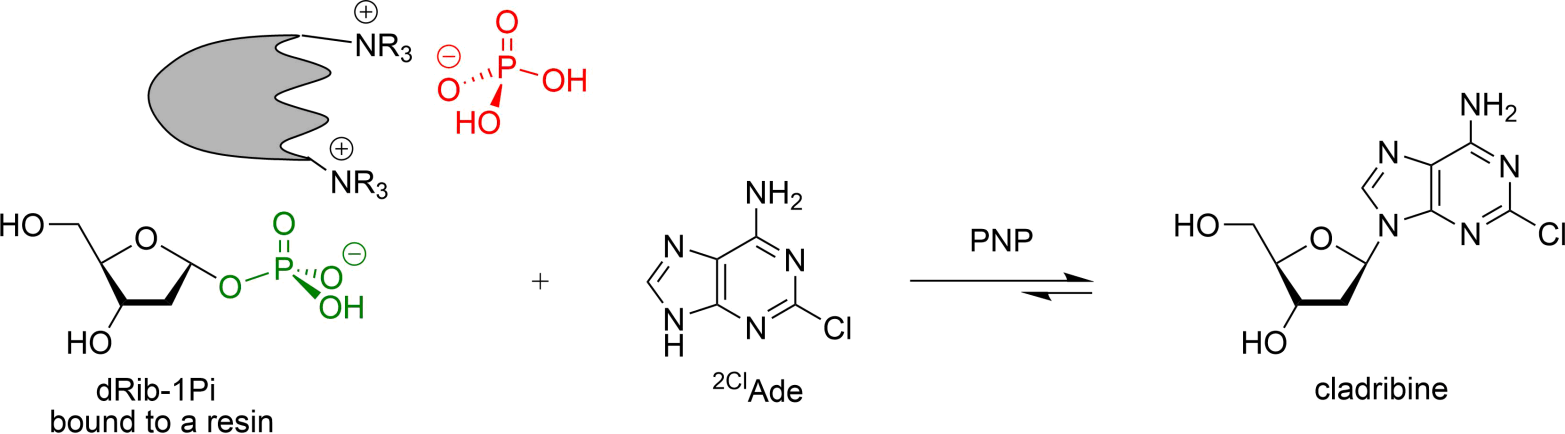	

	HPLC picture in standard reaction conditions

A) Cladribine (standard sample); *t*_R_ 5.30 min; under analogous HPLC analysis the peak corresponding to 2-chloroadenine was at *t*_R_ 3.70 min.	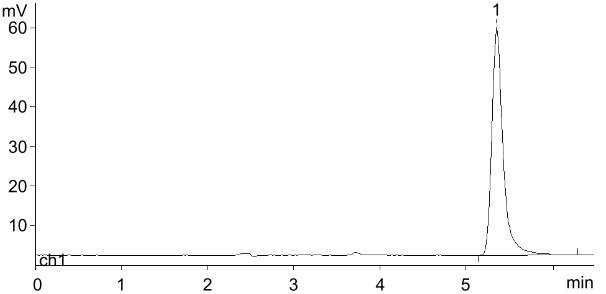
B) The reaction of dRib-1Pi (Ba^2+^) with ^2Cl^Ade [3:1 ratio, mol; in water, catalyzed by *E. coli* PNP (1,800 IU per 1 mmol of base) at 47 °C for 72 h]. (conversion of ^2Cl^Ade → cladribine; 88%; *t*_R_ 3.90 and 5.55 min; ratio 12:88)	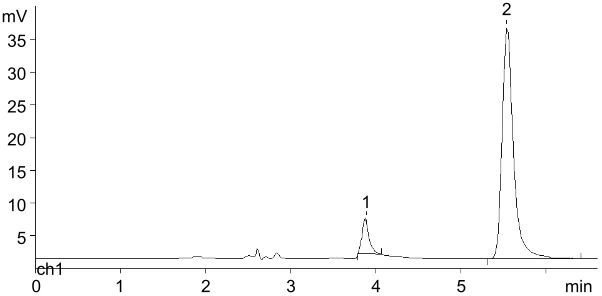
C) The reaction of dRib-1Pi ionically bound by QAE Sephadex A-25-*n*Pi and ^2Cl^Ade catalyzed by *E. coli* PNP (2,288 IU per 1 mmol of base at 40 °C for 96 h. (conversion of ^2Cl^Ade → cladribine; 81%; *t*_R_ 3.73 and 5.36 min; ratio 19:81). Peak at *t*_R_ 2.75 min corresponds to thymine originating from the phosphorolysis of thymidine and adsorbed by the resin.	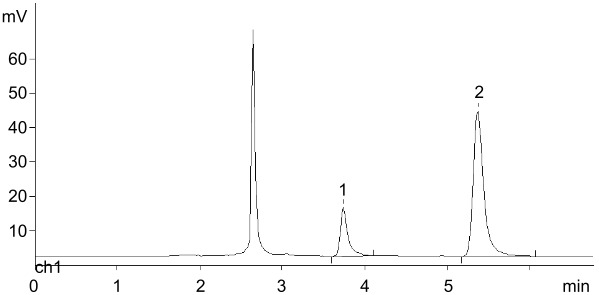

Initially, dRib-1Pi (Ba^2+^) was obtained by the phosphorolysis of thymine in the presence of *E. coli* TP essentially as described above for other PF-1Pis, and its interaction with 2-chloroadenine (^2Cl^Ade) catalyzed by *E. coli* PNP was studied. Work-up of the reaction mixture and purification as described above gave dRib-1Pi (Ba^2+^) in 93% yield. The reaction reached the equilibrium after incubation of the reaction mixture at 47 °C for 72 h with 88% concentration of cladribine. The suggested scheme of the cladribine synthesis appears to be more efficient compared with those published earlier by us [71–72] as well as to other recent reports [25,34,37,73–77] (for a review, see [5]). It is noteworthy that in the pioneering works by Friedkin and Kalckar ([78–79]; see also 17,18]) on the preparation of dRib-1Pi and its reaction with hypoxanthine in phosphate-free medium [Tris·HCl buffer, pH 8.55; at 30 °C for 30 min] they observed a rapid conversion of the base into the corresponding nucleoside.

The complete phosphorolysis of thymidine catalyzed by *E. coli* TP in the presence of QAE Sephadex-*n*Pi and MgCl_2_, separation of the resin with ionically bound dRib-1Pi and its incubation in water milieu in the presence of ^2Cl^Ade and *E. coli* PNP at 40 °C for 96 h resulted in the establishment of equilibrium at 81% conversion of the base into cladribine (Table 2). The reaction mixture was worked-up and the product was purified by silica gel column chromatography to afford cladribine in 59% yield that was identical (HPLC, TLC chromatography, UV, and NMR spectroscopy) to the sample obtained previously [72].

## Conclusion

In the present work we outlined and validated a new approach to the enzymatic synthesis of nucleosides employing nucleoside phosphorylases in combination with anion exchange resins. The results of the presented experiments convincingly demonstrated that anion exchange resins in the phosphate form can serve as a supplier of inorganic phosphate in the reaction of enzymatic phosphorolysis, a PF-1Pi acceptor formed as a result of phosphorolysis, and a PF-1Pi source in the enzymatic synthesis of biologically important nucleosides. The possibilities of the proposed method were illustrated by the synthesis of nelarabine, kinetin riboside and cladribine. A number of anion exchange resins have been tested, and as a result, it was found that QAE Sephadex A-25-nPi can effectively perform two functions, viz., serve as a donor of inorganic phosphate in the phosphorolysis reaction, and also bind PF-1Pi and release it in the nucleoside synthesis. On the contrary, Dowex-*n*Pi can effectively carry out only the first function. The *E. coli* PNP-catalyzed reaction of *N*^6^-benzyladenine and Rib-1Pi bound by the test resin during the phosphorolysis of uridine was used to test the ability of the resin to transfer Rib-1Pi in terms of the amount of *N*^6^-benzyladenosine formed.

It should be noted that the suggested strategy for the enzymatic synthesis of biologically important nucleosides offers a new route to the solution of the problem communicated by Kalckar (vide supra), namely, a route to remove “*all traces of inorganic phosphate*” from the reaction of PF-1Pi and heterocyclic base.

## Experimental

### General methods and materials

All chemicals and solvents were of laboratory grade obtained from commercial suppliers and were used without further purification. Column chromatography (CC) was performed on silica gel 60 H (70–230 mesh ASTM; Merck, Darmstadt, Germany), except where otherwise indicated. TLC was performed on TLC glass plates covered with silica gel 60 F_254_ (Merck, Germany). The TLC control of the PF-1Pis synthesis and conversions was tested first by UV light then by heating of the plates. UV–vis spectra were recorded with a Shimadzu UV-Mini 1240 (Shimadzu, Japan). ^1^H and ^13^C NMR spectra were measured at 500.13 and 125.75 MHz at 300 K on a Bruker Avance-500 spectrometer (Bruker BioSpin GmbH, Germany). δ Values are given in ppm downfield from internal SiMe_4_ (^1^H, ^13^C) (s = singlet; d = doublet; t = triplet; q = quadruplet; m = multiplet; br.s = broad signal); coupling constants *J* are given in Hz. HPLC system: A) HPLC compact pump 2050 with a Lambda 1010 UV detector (Bischoff Chromatography, Germany), column Agilent Eclipse Plus C18 (4.6 × 150 mm, 5 μm), isocratic elution; B) Waters HPLC system (Waters, USA) with UV detector, column Nucleodur EC C18 (250 × 4.6, 100-5 isocratic elution). The following anion exchange resins were used in the present work: (1) Dowex^®^ 1X8 chloride form, 100–200 mesh, exchange capacity 1.2 mequiv/mL by wetted bed volume (Serva, Germany; cat. Number 4110102). (2) QAE Sephadex A-25 chloride form, 40–120 μm, exchange capacity 3 ± 0.4 mequiv/g (Pharmacia Fine Chemicals, USA; cat. Number 17-0170-02). (3) BIO-RAD AG^®^ 1-X2 Anion Exchange Resin chloride form, 200–400 mesh, exchange capacity 3.5 mequiv/g (Bio-Rad, USA; cat. Number 1401251). (4) DEAE-Cellulose, exchange capacity 0.6 mequiv/g (Reanal, Hungary; CAS 9013-34-7). The conversion of the obtained resins to the phosphate form was carried out in the usual manner. A graphical representation and fitting of the data were performed in the online editor Plotly Chart Studio https://plotly.com/chart-studio/

The recombinant *E. coli* thymidine phosphorylase (TP, specific activity 167 IU per mg protein), uridine phosphorylase (UP, specific activity 140 IU per mg protein in solution 17 mg/mL), and purine nucleoside phosphorylase (PNP, specific activity 27 IU per mg of protein) [80] have been used throughout of the studies. The powdered enzymes were dissolved in 5 mM K-phosphate buffer (pH 7.0) at room temperature and then added to the reaction mixtures.

### Synthesis of nelarabine

**Synthesis of α-ᴅ-arabinofuranosyl-1-phosphate as barium salt (Ara-1Pi, Ba^2+^) by phosphorolysis of 1-(β-ᴅ-arabinofuranosyl)uracil (Ara-U) catalyzed by *E. coli* UP**. To a suspension of Dowex-*n*Pi (6 mL; 1.2 mequiv/mL) in 15 mL of distilled water; pH 7.0), Ara-U (170 mg, 0.7 mmol) and MgCl_2_·6H_2_O (71 mg, 0.5 mmol of MgCl_2_ per 1 mmol of Ara-U), followed by UP (105 IU; 150 units per 1 mmol of substrate) were added, and the reaction mixture was gently stirred at 40 °C with monitoring the Ara-U phosphorolysis by TLC (eluent СHCl_3_/MeOH 4:1 (v/v), development of the spots by UV (Ara-U and uracil)) and HPLC (A; elution with 4% MeCN in H_2_O (v/v), 0.7 mL/min; *t*_R_ 2.9 and 5.3 min for uracil and Ara-U, respectively).

After 72 h the complete (>97%) phosphorolysis of Ara-U was observed and the reaction mixture was stored at 4 °C overnight. The Dowex-*n*Pi (adsorbed part of Ara-1Pi formed) and uracil precipitated were filtered off, and an aqueous solution containing Ba(OAc)_2_ (267 mg, 1.05 mmol; 1.5 mmol per 1 mmol of starting Ara-U) and 25% NH_4_OH (5 mL; total volume 15 mL) was added to the filtrate, and the reaction mixture (pH 8.0) was stored at 4 °C overnight.

The precipitate formed was filtered off and the filtrate was evaporated to ca. 15 mL. Then, ethanol (75 mL) was added under stirring and the mixture was stored at 4 °C overnight. The precipitate formed was filtered off, washed with ethanol (2 × 15 mL), dried in vacuum under CaCl_2_ at rt to constant weight to give Ara-1Pi (Ba salt; 244 mg, 96%); TLC: iPrOH/25% NH_4_OH/water 11:2:5 (vol), one spot with *R*_f_ 0.30, development by heating). The ^1^H NMR spectrum was similar to that published earlier [29].

**Synthesis of nelarabine by reaction of Ara-1Pi (Ba^2+^) and *O*^6^-methylguanine (OMG) catalyzed by *E. coli* PNP.** The barium salt of Ara-1P (73 mg, 0.2 mmol) and PNP (280 IU; 1,400 IU and 2,333 per mmol of Ara-1Pi and base, respectively) were added to a solution of OMG (20 mg, 0.12 mmol; dissolved by heating) in distilled water (50 mL) at room temperature. The reaction mixture was gently stirred at 47 °C for 48 h with monitoring the product formation by HPLC (HPLC system A, 5% H_2_O/MeCN), λ = 260 nm, 1 mL/min; *t*_R_ 4.5 and 5.1 min for the base and nucleoside, respectively). After 48 h, the conversion of the base into nucleoside reached 85%. The turbid (not reacted starting base according to TLC and barium phosphate) reaction mixture was filtered, the filtrate evaporated, and the residue treated with EtOH (20 mL). The non-dissolved fine powder was filtered off, the filtrate evaporated and co-evaporated with EtOH (2 × 20 mL). The residue was dissolved in MeOH (10 mL), mixed with silica gel (2 mL) and evaporated and the solid was put on the top of a silica gel column (1.5 × 23 cm) prepared in EtOAc. The fractions containing the nucleoside were combined, evaporated and dried to afford the powdered product (25 mg; 66%) of 95.5% purity (HPLC), that was crystallized from MeCN to give nelarabine (19 mg; 53%; 99.3% purity according to HPLC). UV (5 mM K-phosphate buffer, pH 7.0) λ_max_, nm (ε): 212 (18,150), 249 (9,450), 281 (9,950); λ_min_, nm (ε): 227 (1,950), 260 nm (4,950); UV (H_2_O) λ_max_, nm (ε): 249.1 (9,500), 279.9 (9,970); λ_min_, nm (ε): 226.5 (2,050), 260.9 (2,350); lit. data [43], UV (50 mM phosphate buffer (pH 7.0)/ethanol 9:1, v/v) λ_max_, nm (ε): 247.5 (9,100), 279.0 (9,300); for ^1^H and ^13^C NMR data see [42] and Supporting Information File 1.

**Synthesis of nelarabine employing crude Ara-1Pi without isolation from the reaction mixture of Ara-U phosphorolysis.** Ara-U (45 mg, 0.184 mmol) was dissolved in a suspension of anion exchange resin Dowex-*n*Pi (3 mL of resin in 10 mL of distilled water). Then, MgCl_2_·6H_2_O (41 mg, 0.2 mmol of MgCl_2_ per 1 mmol of Ara-U) and UP (95 IU; 516 units per 1 mmol of substrate) were added, and the reaction mixture kept at room temperature for 72 h (pH 7.0). The progress of the Ara-U phosphorolysis was monitored by TLC (A) and HPLC. HPLC analysis showed that ca. 96% of Ara-U was phosphorolyzed after 72 h (HPLC system A, elution with 4% MeCN in water (v/v); λ = 260 nm, 0.7 mL/min; *t*_R_ 2.9 and 5.25 min for uracil and Ara-U, respectively). The Dowex resin was filtered off, washed with distilled water (2 × 5 mL) and the combined aqueous phase added to a solution of OMG (15 mg, 0.091 mmol) in distilled water (10 mL; dissolved by heating). To the resulting solution at 40 °C, PNP (135 IU; 1,484 per 1 mmol of base) was added and the reaction mixture was gently stirred at 50 °C for 120 h with monitoring of the formation of nelarabine by HPLC: (system A, elution with 4% MeCN in H_2_O (v/v), 1 mL/min; λ = 260 nm, *t*_R_ 12.3 and 19.8 min for heterocyclic base and nelarabine, respectively. After 120 h, the reaction reached equilibrium at ca. 60% base → nelarabine conversion. Work-up of reaction mixture and purification/crystallization as described above gave nelarabine in 52% yield (14 mg; 99.0% purity according to HPLC).

### Synthesis of kinetin riboside

**Synthesis of α-ᴅ-ribofuranosyl-1-phosphate as barium salt (Rib-1Pi, Ba^2+^) by phosphorolysis of uridine catalyzed by *E. coli* UP.** To a suspension of Dowex-*n*Pi (5 mL; 1.2 mequiv/mL) in 10 mL of distilled water; pH 7.0), uridine (63 mg, 0.258 mmol) and MgCl_2_·6H_2_O (11 mg, 0.2 mmol of MgCl_2_ per 1 mmol of uridine) followed by UP (100 IU; 387 units per 1 mmol of substrate) were added and the reaction mixture was gently stirred at rt with monitoring the uridine phosphorolysis by TLC (eluent СHCl_3_/MeOH 4:1 (v/v), development of the spots by UV (uridine and uracil)) and HPLC (A; elution with 4% MeCN in H_2_O (v/v), 0.7 mL/min; *t*_R_ 2.9 and 4.2 min for uracil and uridine, respectively).

After 60 h the complete (>97%) phosphorolysis of uridine was observed and the reaction mixture was stored at 4 °C overnight. The Dowex-1Pi (adsorbed part of Rib-1Pi formed) and precipitated uracil were filtered off, an aqueous solution containing Ba(OAc)_2_ (99 mg, 0.39 mmol; 1.5 mmol per 1 mmol of uridine) and 25% NH_4_OH (2 mL; total volume 15 mL) was added to the filtrate, and the reaction mixture (pH 8.0) was stored at 4 °C overnight. The precipitate formed was filtered off and washed with distilled water. The filtrate was evaporated to ca. 10 mL, ethanol (50 mL) added under stirring, and the mixture stored at 4 °C overnight. The precipitate formed was filtered off, washed with ethanol (2 × 10 mL), dried in vacuum under CaCl_2_ at rt to constant weight to give Rib-1Pi (Ba^2+^); 91 mg, 96%; TLC: iPrOH/25% NH_4_OH/water 7:1:2 (vol), one spot with *R*_f_ 0.17, development by heating). The NMR data were similar to those published earlier [29].

**Synthesis of kinetin riboside (KR) employing the crude Rib-1Pi (Ba^2+^) without isolation from the reaction mixture of uridine phosphorolysis.** The *E. coli* UP (95 IU; 580 units per 1 mmol of uridine) was added to the heterogeneous mixture containing uridine (40 mg, 0.164 mmol), Dowex-*n*Pi (3 mL of wet resin), and MgCl_2_·6H_2_O (40 mg, 0.2 mmol; mmol of MgCl_2_ per 1 mmol of uridine) in distilled water (10 mL). The reaction mixture was kept with gentle mixing at room temperature for 72 h monitoring the product formation by HPLC (HPLC system B, 4% MeCN in H_2_O, λ = 260 nm, 0.7 mL/min; *t*_R_ uracil, 6.2 min; uridine, 8.5 min). Under these conditions, the complete (>97%) phosphorolysis of uridine was observed. The Dowex resin was filtered off, kinetin (17 mg, 0.079 mmol) and *E. coli* PNP (27 IU; 342 units per 1 mmol of kinetin) were added to the filtrate, and the reaction mixture was gently stirred at 50 °C for 144 h giving rise to the base → nucleoside conversion of 72% according to HPLC analysis (HPLC system A, 17% MeCN in water (v/v). *t*_R_ values: kinetin, 11.4; nucleoside, 15.5 min). Conventional work-up followed by silica gel column (1.7 × 25 cm) chromatography afforded 17 mg (61% yield based on the kinetin amount) KR as yellowish powder of 98.0% purity (HPLC). UV (MeOH) λ_max_, nm (ε): 266 (18,675), 211 (23,900); λ_min_, nm (ε): 233 nm (3,340); ^1^H NMR (500 MHz, DMSO-*d*_6_) δ (purine numbering) purine: 8.41 (s, 1H, H-8), 8.37 (s, 1H, H-2), 8.27 (br.s, 1H, C6-N**H**); furfuryl radical: 7.56 (d, *J* = 0.9 Hz, 1H), 6.38 (dd, *J* = 3.1, 1.8 Hz, 1H), 6.25 (d, *J* = 2.9 Hz, 1H), 4.71 (s, 2H, N-C**H_2_**-); ribose fragment: 5.91 (d, *J* = 6.1 Hz, 1Н, Н-1′), 5.49 (d, *J* = 6.1 Hz, 1H, ОН-2′), 5.40 (dd, *J* = 6.9, 4.7 Hz, 1H, ОН-5′), 5.23 (d, *J* = 4.5 Hz, 1H, ОН-3′), 4.63 (dd, *J* = 11.1, 5.8 Hz, 1Н, Н-2′), 4.17 (dd, *J* = 7.5, 4.2 Hz, Н-3′), 3.98 (q, *J* = 3.5 Hz, Н-4′), 3.69 (dt, *J* = 11.9, 4.0 Hz, Н-5′), 3.57 (3.61–3.53, m, 1Н, Н-5′′) ppm. For ^1^H NMR spectrum (500 MHz, MeOD), see Supporting Information File 1; ^13^С NMR (DMSO-*d*_6_); purine: 152.87 (C2), 148.50 (C4), 119.5 (C5), 154.32 (C6), 140.01 (C8); ribose fragment: 87.90 (C1′), 73.48 (C2′), 70.61 (C3′), 85.86 (C4′), 61.63 (C5′); furfuryl fragment: 141.81 (correlation with the proton resonance at 7.56 ppm), 110.43 (correlation with the proton resonance at 6.38 ppm), 106.63 (correlation with the proton resonance at 6.25 ppm), the C1-*ipso* at 152.87 and the *C*H_2_ resonance at 36.53 ppm (cf. with the ^1^H and ^13^C NMR data in CD_3_OD [60]). See also Supporting Information File 1.

**Synthesis of kinetin riboside by the reaction of Rib-1Pi (Ba^2+^) and kinetin catalyzed by *E. coli* PNP in Tris·HCl buffer.** Kinetin (15 mg, 0.07 mmol) and α-ᴅ-ribofuranose-1-phoshate (Ba^2+^) (50 mg, 0.14 mmol; Rib-1Pi:base 2:1, mol) were dissolved in Tris·HCl buffer (50 mM, 40 mL, pH 8) and PNP (18.4 IU; 263 units per 1 mmol of base) was added to the reaction mixture that was kept at room temperature with gentle stirring. The progress of the reaction was monitored by HPLC (system A, 17% aq. MeCN, *t*_R_ 11.4 and 15.5 min for kinetin and KR, respectively). After 2 h, almost complete transformation of the base into the nucleoside was observed according to HPLC. The reaction mixture was evaporated to dryness, the residue dissolved in ethanol, absorbed on silica gel (2 mL), and put on the top of a silica gel column (1.5 × 23 cm), which was then eluted with chloroform/methanol 9:1→8:1 (v/v) to afford KR as yellowish powder (22.5 mg, 93%, based on the kinetin amount; purity 98.5% according to HPLC).

### Synthesis of cladribine

**Synthesis of dRib-1Pi (Ba^2+^) and its reaction with 2-chloroadenine catalyzed by *E. coli* PNP affording cladribine**. To a solution of thymidine (165 mg, 0.68 mmol) in 10 mL of distilled water were added Dowex-*n*Pi (20 mL, pH 7.0), MgCl_2_·6H_2_O (70 mg, 0.35 mmol per 1 mmol of substrate), and *E. coli* TP (45 IU; 66 units per 1 mmol of substrate), and the mixture was gently stirred at 40 °C for 120 h resulting in the complete phosphorolysis of the starting thymidine (>97%) according to HPLC analysis (A; elution with 4% MeCN in water (v/v), 0.7 mL/min; *t*_R_ 5.4 and 12.6 min for thymine and thymidine, respectively). The reaction mixture was worked up as described above for the preparation of Ara-1Pi, and the filtrate was kept in a refrigerator at 4 °C for ≈15 h. The precipitate formed was filtered off and the filtrate was mixed with a water/ammonia (1 mL 25% NH_4_OH + 5 mL distilled water) solution of Ba(OAc)_2_ (226 mg, 1.3 mmol per 1 mmol of thymidine), and the resulting mixture was stored at 4 °C overnight (pH 8). The precipitate formed was centrifuged off and the supernatant evaporated under vacuum to a volume of ca.10 mL. To the concentrated solution was added dropwise under stirring ≈5 volumes of ethanol and the mixture kept overnight in the refrigerator. The precipitate formed was filtered off, washed with ethanol (2 × 20 mL), dried in vacuum under CaCl_2_ at rt for two days to give dRib-1Pi (Ba^2+^). White powder, 220 mg, 93%; TLC: iPrOH/25% NH_4_OH/water 7:1:2 (vol), one spot with *R*_f_ 0.30). The ^1^H and ^13^C NMR spectra in DMSO-*d*_6_ of this product were consistent with literature data [28].

The reaction of dRib-1Pi (Ba^2+^) (32 mg, 0.09 mmol) with 2-chloroadenine (5 mg, 0.03 mmol) in aqueous solution in the presence of *E. coli* PNP (1,800 IU per 1 mmol of heterobase) at 47 °C for 72 h reached equilibrium at 88% (conversion) concentration of cladribine in the reaction mixture according to HPLC analysis data.

**The reaction of dRib-1Pi ionically bound with QAE Sephadex A-25 (phosphate form) and ^2Cl^Ade catalyzed by *E. coli* PNP.** The reaction mixture containing thymidine (135 mg, 0.56 mmol), MgCl_2_·6H_2_O (38 mg, 0.2 mmol per 1 mmol of thymidine), *E. coli* TP (50 IU, 89 units per 1 mmol of thymidine), and QAE Sephadex-*n*Pi (30 mL; total volume 40 mL; pH 7.20) was stirred at 40 °C for 120 h giving rise to complete phosphorolysis of the starting thymidine (>97%) according to HPLC analysis. The resin with absorbed dRib-1Pi formed was filtered off, washed with distilled water (3 × 30 mL) and added to a solution (40 mL) of ^2Cl^Ade (10 mg, 0.059 mmol) and *E. coli* PNP (135 IU, 2,288 units per base), and the reaction mixture was gently stirred at 40 °C with monitoring cladribine formation by HPLC. After 96 h, the reaction achieved equilibrium at 81% concentration of cladribine. The enzyme was deactivated by short heating in boiling water, the mixture was cooled and kept in a freezer at 4 °C overnight. The resin, containing ^2Cl^Ade (HPLC) and thymine, was washed with MeOH/H_2_O 1:1 (vol; 10 mL), the combined filtrate evaporated to dryness and co-evaporated with ethanol. The residue was dissolved in ethanol, absorbed on silica gel (2 mL), and put on the top of the silica gel column (2 × 19 cm), which was then eluted with 5 → 20% MeOH/CHCl_3_ (v/v) to afford cladribine (10 mg, 59%) that was identical with a standard sample. The ^1^H and ^13^C NMR spectra in DMSO-*d*_6_ of this product were consistent with literature data [81].

## Abbreviations

**Table 3:** Abbreviations

Abbreviation	Explanation

UP	uridine phosphorylase
TP	thymidine phosphorylase
PNP	purine nucleoside phosphorylase
PF-1P and PF-1Ps	α-ᴅ-pentofuranose-1-phosphate(s)
Gua and Guo(dGuo)	guanine and guanosine(2'-deoxyguanosine), respectively
Ura and Urd(dUrd)	uracil and uridine(2'-deoxyuridine), respectively
Ara-U	1-(β-ᴅ-arabinofuranosyl)uracil
Thy and Thd(dThd)	thymine and thymidine(2'-deoxythymidine), respectively
Ino and dIno	inosine and 2'-deoxyinosine
Ade and Ado(dAdo)	adenine and adenosine(2'-deoxyadenosine), respectively
*N*^7^Me-Gua and -Guo(-dGuo)	*N*^7^-methylguanine and -guanosine(-2'-deoxyguanosine), respectively
Rib-1P. dRib-1P and Ara-1P	α-ᴅ-ribo-, 2'-deoxyribo- and arabinofuranose-1-phosphates
KR	kinetin riboside
IU	international units for activity of enzymes
OMG	*O*^6^-methylguanine (OMG)
BAP	*N*^6^-benzylaminopurine
BAPR	*N*^6^-benzylamino-9-(β-ᴅ-ribofuranosyl)purine
^2Cl^Ade	2-chloroadenine

## Supporting Information

File 1 Experimental details and NMR spectra of synthesized compounds.
