# Supplementary material for: Anion exchange resins in phosphate form as versatile carriers for the reactions catalyzed by nucleoside phosphorylases
Source: Beilstein J Org Chem. 2020 Oct 22;16:2607–22. doi: 10.3762/bjoc.16.212 (PMC7588730; doi:10.3762/bjoc.16.212)
Supplement: File 1 — Experimental details and NMR spectra of synthesized compounds. [file Beilstein_J_Org_Chem-16-2607-s001.pdf]

BEILSTEIN JOURNAL OF ORGANIC CHEMISTRY

Supporting Information

for

Anion exchange resins in phosphate form as versatile carriers for the reactions catalyzed by nucleoside phosphorylases

Julia N. Artsemyeva, Ekaterina A. Remeeva, Tatiana N. Buravskaya, Irina D. Konstantinova, Roman S. Esipov, Anatoly I. Miroshnikov, Natalia M. Litvinko and Igor A. Mikhailopulo

Beilstein J. Org. Chem. **2020,** *16,* 2607–2622. doi:10.3762/bjoc.16.212

Experimental details and NMR spectra of synthesized compounds

License and Terms: This is a supporting information file under the terms of the Creative Commons Attribution License (https://creativecommons.org/licenses/by/4.0). Please note that the reuse, redistribution and reproduction in particular requires that the authors and source are credited.
The license is subject to the *Beilstein Journal of Organic Chemistry* terms and conditions: (https://www.beilstein-journals.org/bjoc)

Table of Contents

1 – **Table SI-1**: ^{1}H NMR spectral data for nelarabine, kinetin riboside,

and *N*^{6}-benzyladenosine. S2

2 – **Table SI-2**: ^{13}C NMR spectral data for of nelarabine, kinetin riboside

and *N*^{6}-benzyladenosine. S3

3 – **SI-3**. ^{1}H NMR spectrum of nelarabine S4

4 – **SI-4**. ^{1}H and ^{13}C NMR spectra of kinetin riboside S5

5 – **SI-5**. Synthesis of *N*^{6}-benzyladenoside and ^{1}H and ^{13}C NMR spectra S7

6 – **SI-6**. Combined data on the enzymatic synthesis of nelarabine, kinetin riboside,

N^{6}-benzyladenosine and cladribine.S S10

References...S12

1 – Table SI – 1. ^{1}H NMR spectral data for nelarabine, kinetin riboside, *N*^{6}-benzyladenosine, and cladribine in DMSO-d_6 (δ_{TMS}, ppm).

Compound	*H-1′*	*H-2′* (lower field resonance)	*H-2″* (higher field resonance)	*H-3′*	*H-4′*	*H-5′*	*H-5″*	Others
Nelarabine	6.12 d	-	≈4.06 m	≈4.08	3.75 "q"	3.65 ddd	3.61 ddd	7.92 (s, H-8); 6.42 (s, 2H, N^2H_2);<br>5.60 (d, 1H, *J* = 5.36, 2′-O**H**);<br>5.49 (d, 1H, *J* = 3.48, 3′-O**H**);<br>5.05 (t, 1H, *J* = 5.29, 5′-O**H**)<br>3.96 (s, 3H, OC$\boldsymbol{H}_3$)
Kinetin Riboside	5.96 d	4.68 dt	-	4.21 m	4.03 q	3.74 dt	3.62 ddd	8.45. (s, H-8); 8.31 (s, H-2)<br>7.60 (q, *J* = 0.7 & 1.65 Hz), 6.42 (q, *J* = 1.8 & 3.15 Hz),) & 6.29 (d, *J* = 3.0 Hz); (FF group)<br>8.38 (br. s, 6-NH)<br>5.50 (d, $J_{2',OH}$ = 6.20); 5.42 (q, $J_{5',OH}$ = 4.6 & 7.05; 5′-O**H**); 5.24 (d, $J_{3',OH}$ = 4.70; 3′-O**H**)<br>4.76 (br. s, 2H, -C$\boldsymbol{H}_2$-Ph)
N^{6}-Benzyl-adenosine	5.89 d	4.61 "d"	-	4.15 br. m	3.96 q	3.67 dt	3.55 ddd	8.38. (s, H-8); 8.20 (s, H-2)<br>8.45 (br. s, 6-NH)<br>7.33 (d, ortho), 7.28 (td, meta) & 7.20 (tt, para); (Bzl)<br>5.43 (d, $J_{2',OH}$ = 6.20); 5.42 (q, $J_{5',OH}$ = 4.6 & 7.05; 5′-OH); 5.17 (d, $J_{3',OH}$ = 4.65; 3′-O**H**)<br>4.71 (br. s, 2H, -C$\boldsymbol{H}_2$-Ph)
Cladribine	6.38 d	2.66 ddd	2.30 ddd	4.45-4.36 m	3.88 dt	3.62 ddd	3.53dt	8.38 (s, H-8); 7.85 (br.s, NH_2)<br>5.34 (d, *J* = 4.2 Hz; C3′-O**H**)<br>4.98 (t, *J* = 5.6 Hz; C5′-O**H**)

2 – Table SI – 2. ^{13}C NMR spectral data for nelarabine, kinetin riboside, *N*^{6}-benzyladenosine, and cladribine in DMSO-d_6 (δ_{TMS}, ppm)**.**

Compound	Chemical shifts (δ_{TMS}, ppm)									
	C1'	C2'	C3'	C4'	C5'	C2	C4	C5	C6	C8
Nelarabine [a])	84.20<br>(83.35)	75.47<br>75.29	75.29<br>75.47	83.35<br>(84.20)	60.95	153.98	159.74	113.16	160.52	139.06
Kinetin Riboside [b])	87.90	73.48	70.61	85.86	61.63	152.87	148.50	≈119.5	154.32	140.01
N^6-Benzyladenosine [c,d])	87.93	73.45	70.64	85.88	61.65	152.33	148.45	119.78	154.50	139.94
Cladribine	84.04	e)	71.19	88.43	62.14	157.44	150.55	118.65	157.28	140.30

a) O***C***H_3 54.05 ppm.

b) The ^{13}C resonances of KR (δ_{TMS}, ppm): 141.81 [correlates with a proton (q, *J*'s = 0.70 and 1.65 Hz) at 7.60 ppm]; 110.43 [correlates with a proton (q, *J*'s = 1.80 and 3.15 Hz) at 6.42 ppm]; 106.63 (correlates with a proton (d, *J* = 3.0 Hz) at 6.29 ppm]; the C1-*ipso* of the FF ring appeared at 152.87 ppm and the ***C***H_2 resonance is at 36.53 ppm.

c) The ^{13}C resonances of BzlNH (δ_{TMS}, ppm): 140.09 (*ipso*), 128.20 (*ortho*), 127.12 (*metha*), 126.60 (*para*), and 42.86 (***C***H_2).

d) The ^{13}C-*ipso* resonance of the Bzl group is overlapped by the ^{13}C-8 resonance.

e) The resonance signal of C-2' is overlapped by DMSO

3 – **SI-3**. ^{1}H NMR spectrum of nelarabine in MeOD.

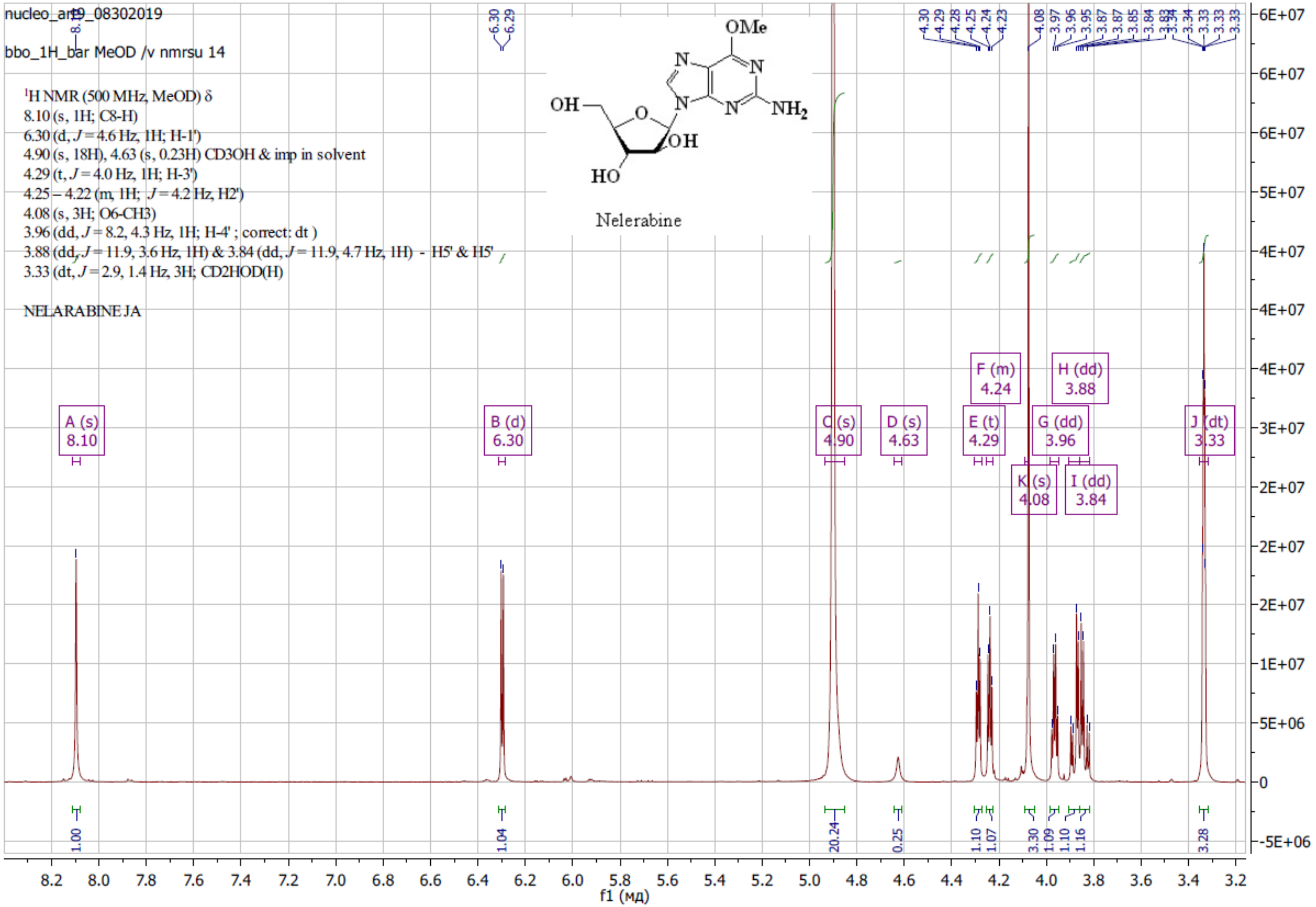

4 – SI-4. ^{1}H and ^{13}C NMR spectra of kinetin riboside in CD_5OD.

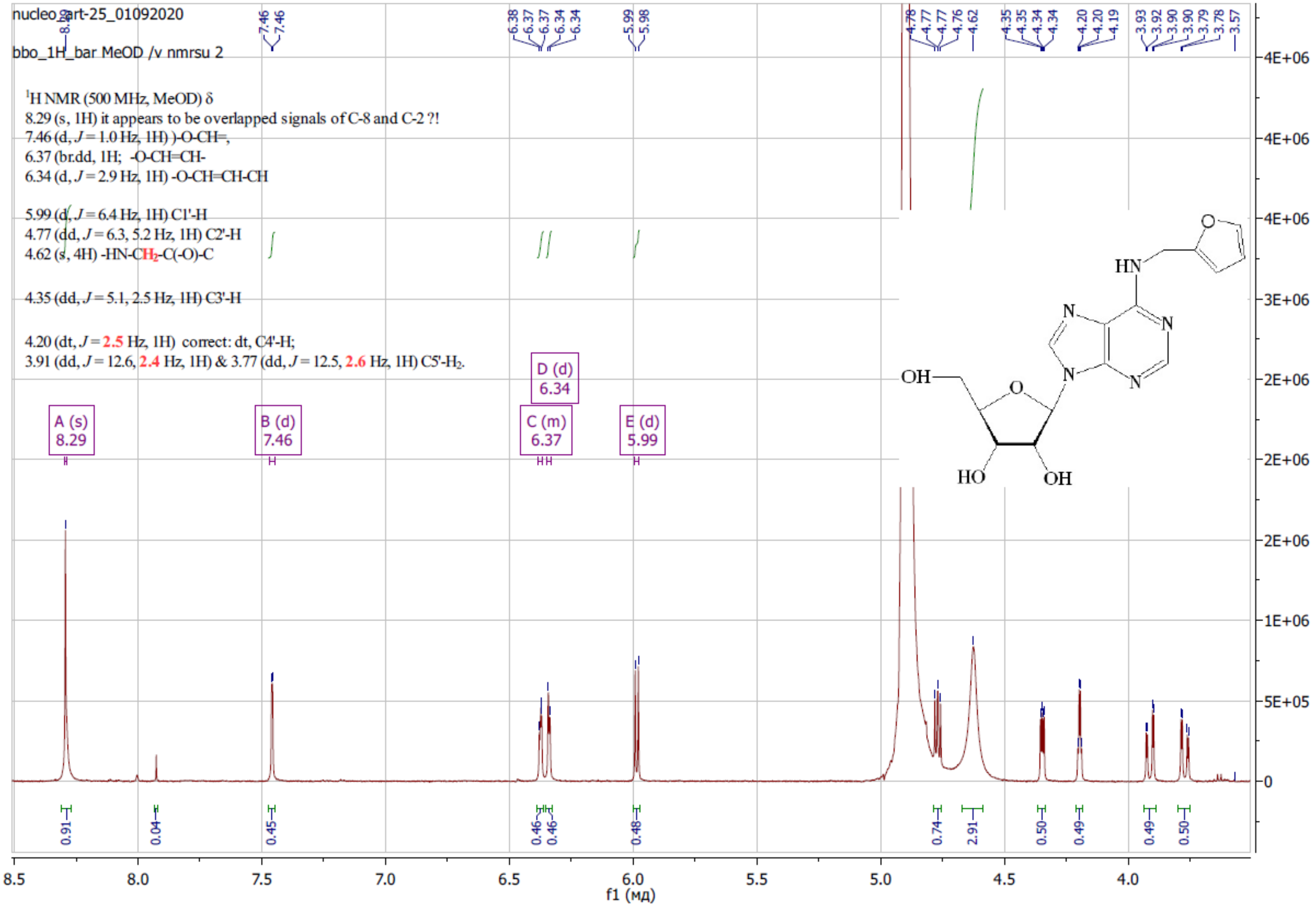

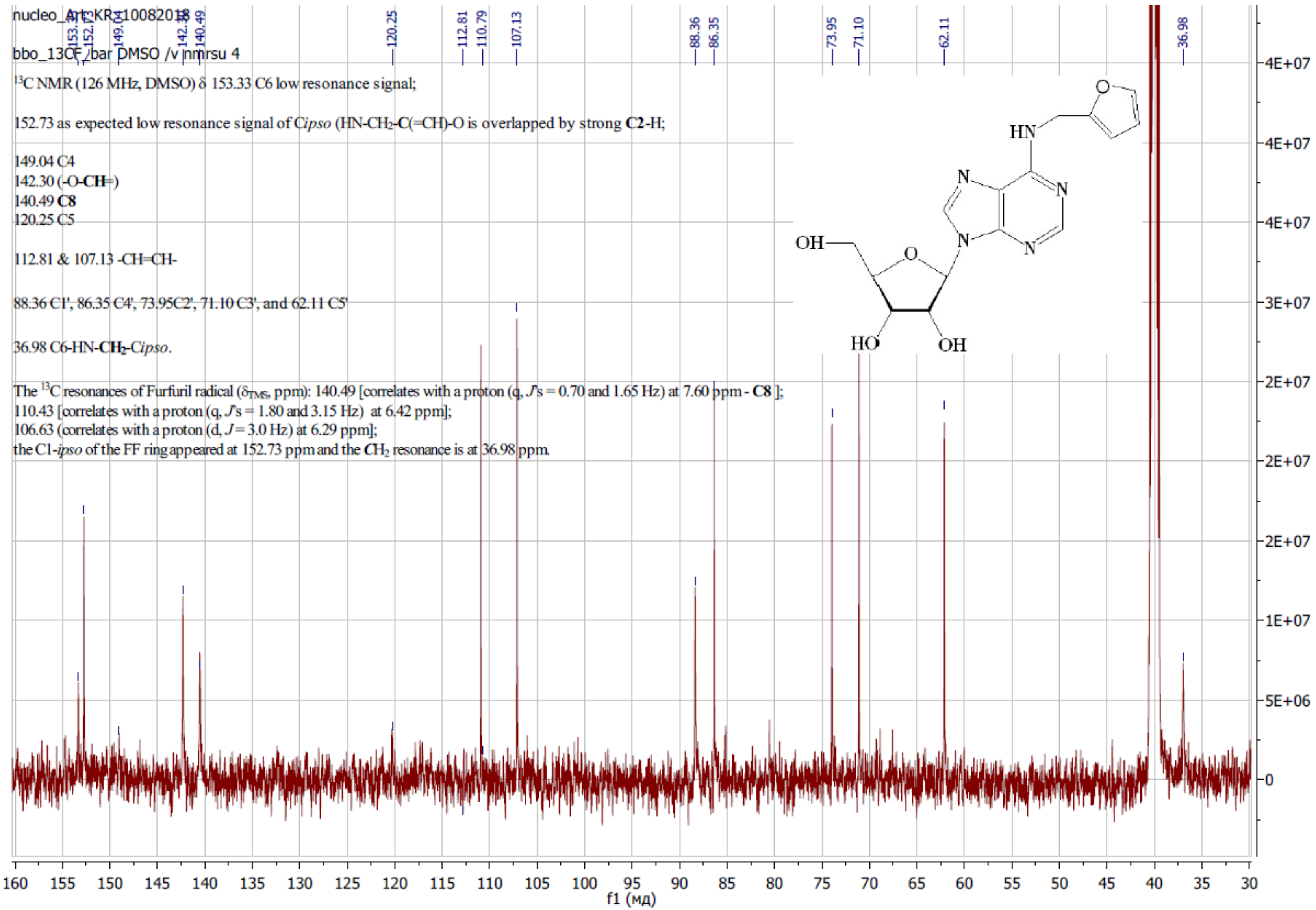
bbo_13CF_bar DMSO /v nmrsu 4
120.25
112.81
110.79
107.13
88.36
86.35
73.95
71.10
62.11
36.98
^{13}C NMR (126 MHz, DMSO) δ 153.33 C6 low resonance signal;
152.73 as expected low resonance signal of C*ipso* (HN-CH$_2$-**C**(=CH)-O is overlapped by strong **C2**-H;
149.04 C4
142.30 (-O-**CH**=)
140.49 **C8**
120.25 C5
112.81 & 107.13 -CH=CH-
88.36 C1', 86.35 C4', 73.95C2', 71.10 C3', and 62.11 C5'
36.98 C6-HN-**CH$_2$**-C*ipso*.
The ^{13}C resonances of Furfuril radical (δ_{TMS}, ppm): 140.49 [correlates with a proton (q, *J*'s = 0.70 and 1.65 Hz) at 7.60 ppm - **C8**];
110.43 [correlates with a proton (q, *J*'s = 1.80 and 3.15 Hz) at 6.42 ppm];
106.63 (correlates with a proton (d, *J* = 3.0 Hz) at 6.29 ppm];
the C1-*ipso* of the FF ring appeared at 152.73 ppm and the **C**H$_2$ resonance is at 36.98 ppm.
HN
O
N
N
N
N
OH
O
HO
OH
4E+07
3E+07
2E+07
1E+07
5E+06
0
160 155 150 145 140 135 130 125 120 115 110 105 100 95 90 85 80 75 70 65 60 55 50 45 40 35 30
f1 (мд)

5 – **SI-5**. Synthesis and ^{1}H and ^{13}C NMR spectra of N^6-benzyladenoside in DMSO-d_6.

***N*^{6}-Benzyladenosine**: To a heterogeneous reaction mixture containing guanosine (425 mg, 1.5 mmol) and N^6-benzyladenine (225 mg, 1 mmol) in 140 mL of 5.0 mM K-phosphate buffer (pH 7.0) was added *E. coli* PNP (140 IU) and the mixture was gently stirred at 40 °C for 20 h (some quantity of the starting heterocyclic base remained in the reaction mixture according to TLC). Then, guanosine (0.14 g, 0.5 mmol) was added and the heating continued for 6 h and the reaction mixture was left overnight at 4 °C. The solution was decanted from the precipitate formed and evaporated to dryness, the residue was mixed with one tee spoon of SiO_2 and put on the top of a standard column (2 × 22 cm) packed in DCE/MeOH 95:5 (vol). Elution with the same mixture of solvents gave 20 mg of N^6-benzyladenine and its riboside (0.22 g, yield 67%; purity 99.2% according to HPLC); mp 178-180 °C (from EtOH); UV (H_2O): λ_{max} 269 and 209 nm (ε 20,140 and 25,230), λ_{min} 232 nm (ε 2,720). λ_{max} 269 nm (ε 20,140); lit. data: (*1*) mp 182-183 °C (from EtOH); UV (EtOH), λ_{max} = 271 nm, (ε 4,290) [1]; (*2*) mp 177-179 °C (from EtOH)}. UV (MeOH): λ_{max} 268 nm (ε 20,850); λ_{min} 233 nm (ε 2,570) [2].

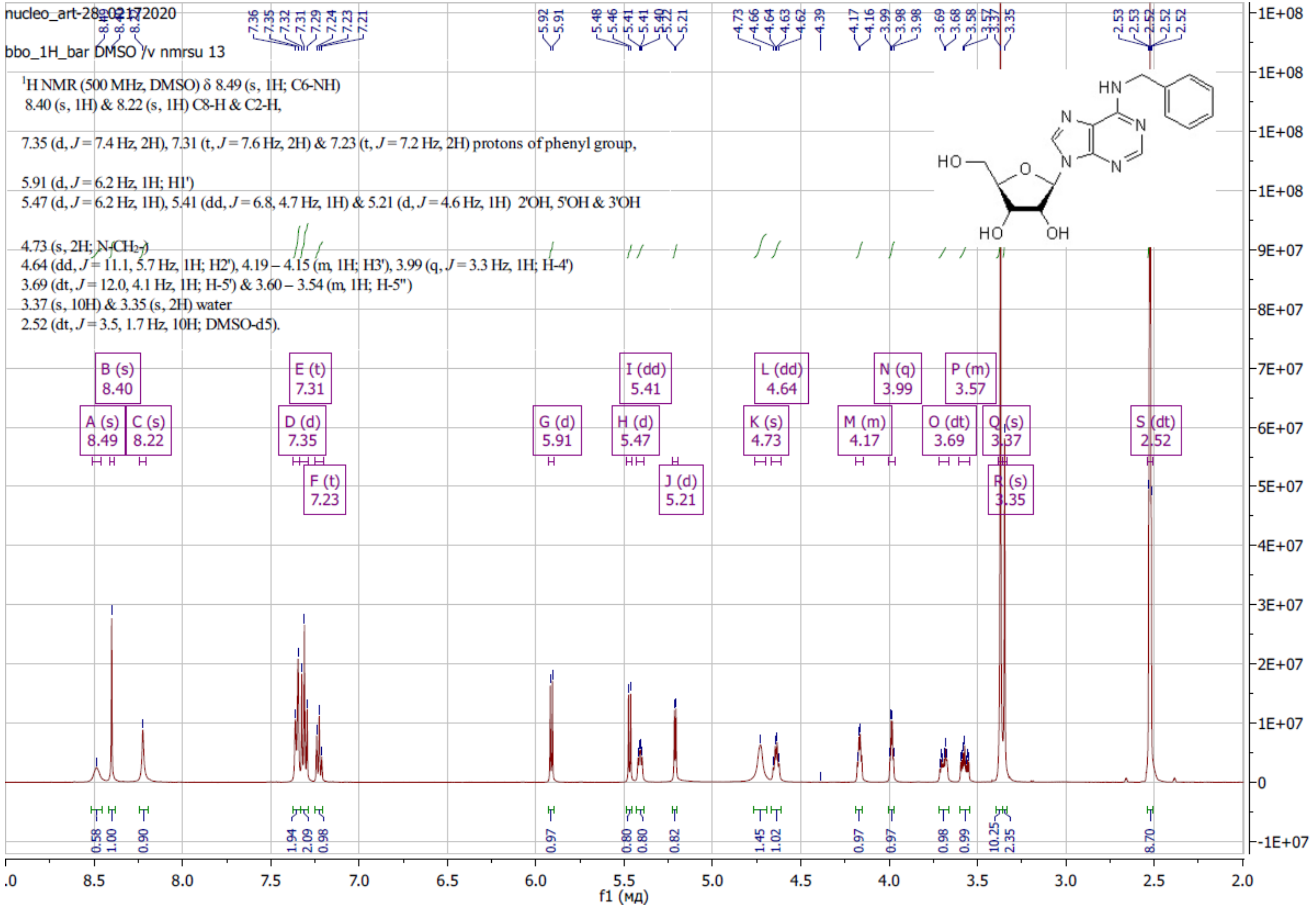
nucleo_art-28022020
bbo_1H_bar DMSO /v nmrsu 13
1H NMR (500 MHz, DMSO) δ 8.49 (s, 1H; C6-NH)
8.40 (s, 1H) & 8.22 (s, 1H) C8-H & C2-H,
7.35 (d, J = 7.4 Hz, 2H), 7.31 (t, J = 7.6 Hz, 2H) & 7.23 (t, J = 7.2 Hz, 2H) protons of phenyl group,
5.91 (d, J = 6.2 Hz, 1H; H1')
5.47 (d, J = 6.2 Hz, 1H), 5.41 (dd, J = 6.8, 4.7 Hz, 1H) & 5.21 (d, J = 4.6 Hz, 1H) 2'OH, 5'OH & 3'OH
4.73 (s, 2H; N-CH2-)
4.64 (dd, J = 11.1, 5.7 Hz, 1H; H2'), 4.19 – 4.15 (m, 1H; H3'), 3.99 (q, J = 3.3 Hz, 1H; H-4')
3.69 (dt, J = 12.0, 4.1 Hz, 1H; H-5') & 3.60 – 3.54 (m, 1H; H-5")
3.37 (s, 10H) & 3.35 (s, 2H) water
2.52 (dt, J = 3.5, 1.7 Hz, 10H; DMSO-d5).
HN
N
N
N
N
HO
O
HO
OH
A (s) 8.49
B (s) 8.40
C (s) 8.22
D (d) 7.35
E (t) 7.31
F (t) 7.23
G (d) 5.91
H (d) 5.47
I (dd) 5.41
J (d) 5.21
K (s) 4.73
L (dd) 4.64
M (m) 4.17
N (q) 3.99
O (dt) 3.69
P (m) 3.57
Q (s) 3.37
R (s) 3.35
S (dt) 2.52
0.58 1.00 0.90 1.94 2.09 0.98 0.97 0.80 0.80 0.82 1.45 1.02 0.97 0.97 0.98 0.99 10.25 2.35 8.70
f1 (мд)

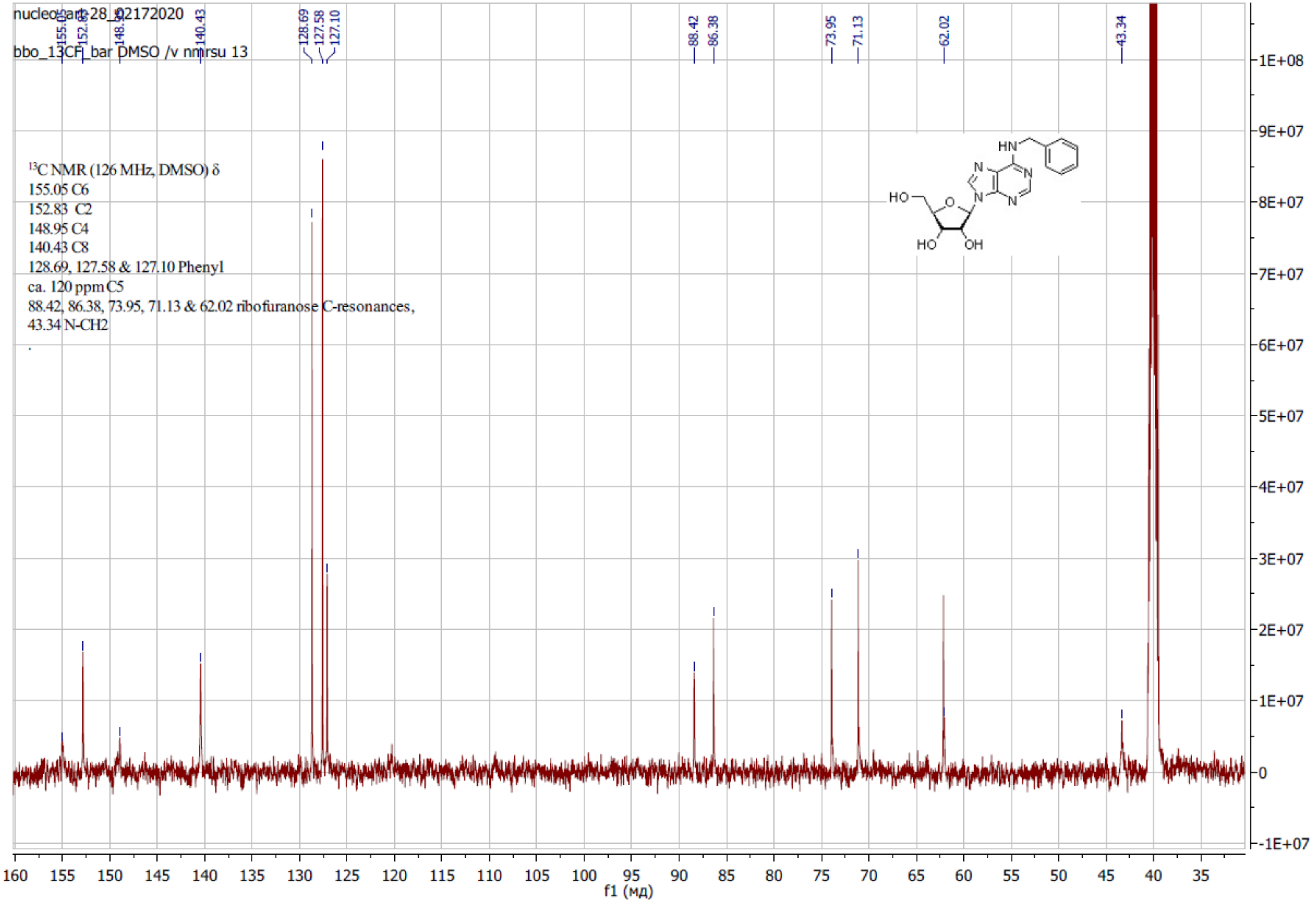
bbo_13CF_bar DMSO /v nmrsu 13
128.69
127.58
127.10
140.43
88.42
86.38
73.95
71.13
62.02
43.34
13C NMR (126 MHz, DMSO) δ
155.05 C6
152.83 C2
148.95 C4
140.43 C8
128.69, 127.58 & 127.10 Phenyl
ca. 120 ppm C5
88.42, 86.38, 73.95, 71.13 & 62.02 ribofuranose C-resonances,
43.34 N-CH2
HN
N
N
N
N
HO
O
HO
OH
1E+08
9E+07
8E+07
7E+07
6E+07
5E+07
4E+07
3E+07
2E+07
1E+07
0
-1E+07
160 155 150 145 140 135 130 125 120 115 110 105 100 95 90 85 80 75 70 65 60 55 50 45 40 35
f1 (мд)

6 – SI-6. Combined data on the enzymatic synthesis of nelarabine, kinetin riboside, N^6-benzyladenosine, and cladribine.

№	Product	Substrate(s)	Enzyme(s) (IU per 1 mmol of substrate)	Reactions condifions		Conver-sion (%)	Yield of pure product (%)	Refs
				Solution	Time (hrs) (Temp.;ºC)			
1	Fludarabine	2F-Ade & Ara-C (1:1.4, mmol)	*E. coli* PNP (93.3) & cytidine deaminase 10 IU *E. coli* UP (8.7)	4 mM Phosphate buffer (pH 7.0)	864 (35)	-	14	[3]
2	Fludarabine	Ara-1Pi & 2F-Ado	*E. coli* PNP (360)	Water	3 (55)	94	77	[4]
3	Nelardbine	Ara-U & C^6OMe-Guo (2:1, mol)	*E. coli* PNP (37,778) *E. coli* UP (1,889)	10 mM Phosphate buffer (pH 7.4)	18 (60)	-	53 (for base taken in the reaction)	[5]
4	Nelarabine	Ara-U & C^6OMe-Guo (1.1:1, mol)	*E. coli* PNP (18,000) *E. coli* UP (1,020)	10 mM *K*-Pi buffer/n-PrOH (7% v/v; (pH 6.75)	624 (37)	-	17	[6]
5	Nelarabine	Chem.Ara-1Pi & C^6OMe-Guo (ca. 5:1, mol)	*E. coli* PNP (49)	Water	36 (45)	50	40	[4]
6	Nelarabine	Ara-1Pi & C^6OMe-Guo (ca. 1.7:1, mol)	*E. coli* PNP (ca. 1,400 prt Ara-1P & 2,333 per base)	Water	48 (47)	85	53 (HPLC purity 99.3%)	This study
7	Nelarabine (test experiment)	Ara-U & C^6OMe-Guo (1.5:1, mol)	*E. coli* PNP (10,000) *E. coli* UP (5,000)	5 mM *K*-Pi buffer (pH 8.00)	1 (23)	Base to nucleoside **44%** Ara-U to Ara-1Pi **≥95**	-	This study
8	Nelarabine	Crude Ara-1Pi & C^6OMe-Guo (ca. 2:1, mol)	*E. coli* UP (516 per Ara-U) *E. coli* PNP (1,484 per base)	Water	1st 72 (r.t.) 2nd 120 (50-55)	1st >95-97% 2nd *ca.* 60%	52 (HPLC purity 99%)	This study
9	Ara-1Pi	Ara-U (170 mg, 0.7 mmol)	*E. coli* UP (150)	Water	72 (40)	**≥97**	96	This study
10	Rib-1Pi	Uridin (63 mg, 0.258 mmol)	*E. coli* UP (387)	Water	60 (23)	**≥97**	96	This study
11	Kinetin Riboside Not optimized!	Crude Ara-1Pi & C^6OMe-Guo (ca. 2:1, mol)	*E. coli* UP (580) >95-97% *E. coli* PNP (342)	Water	1st 72 (r.t.) 2nd 144 (50)	1st >95-97% 2nd 72%	61 (HPLC purity 98%)	This study

12	Kinetin Riboside	Ara-1Pi & C^6OMe-Guo (ca.2:1, mol)	*E. coli* PNP (263)	Tris HCl 50 mM	2 h (r.t.)	>95-97%	93% (HPLC purity 98.5%)	This study
13	*N*^{6}-Benzyl-adenosine	Guanosine (1.5 + 0.5 mmol) & *N*^{6}-^{Bz}Ade (1 mmol)	*E. coli* PNP (120 IU; 80 & 120 units per Guo and base, respectively)	5 mM *K*-Pi buffer (pH 7.00)	20 + 6 h (40)	ca. 10% of base was recovered	67% (HPLC purity 99.2%)	This study
14	Cladribine	Dowex-*n*Pi dRib-1Pi<br>--------------<br>dRib-1Pi & ^{2Cl}Ade (3: 1, mol)	*E. coli* UP (66.0) >95-97%<br>-------------------<br>*E. coli* PNP (1,800 per base)	Water	120 h (40 ºC)<br>-----------------<br>72 h (47 ºC)	>95-97%<br>------------<br>88%	93%<br>------------<br>-	This study
15	Cladribine	Thd (0.56 mmol) QAE Sephadex-*n*Pi & dRib-1Pi<br>+ ^{2Cl}Ade (0.059 mmol)	*E. coli* UP (89.0) >95-97%<br>-------------------<br>*E. coli* PNP (2,288 per base)	Water	120 h (40 ºC)<br>-----------------<br>96 h (40 ºC)	>95-97%<br>------------<br>81%	----------<br>59%	This study

References and notes

1. Zinchenko, A. I.; Barai, V. N.; Lyakhovets, V. I.; Kulak, T. I.; Kvasyuk, E. I.; Mikhailopulo, I. A. *Russ. J. Bioorg. Chem.* **1988,** *14*, 1401–1404.
2. Kissman, H. M.; Weiss, M. J. *J. Org. Chem.* **1956,** *21*, 1053–1055. doi:10.1021/jo01115a621
3. Koszalka, G.W.; Averett, D.R.; Fyfe, J.A.; Roberts, G.B.; Spector, T.; Biron, K.; Krenitsky, T.A. *Antimicrob Agents Chemother*. **1991**, *35*(7), 1437-1443; doi: 10.1128/aac.35.7.1437
4. Konstantinova, I.D.; Antonov, K.V.; Fateev, I.V.; Miroshnikov, A.I.; Stepchenko, V.A.; Baranovsky, A.V.; Mikhailopulo, I.A. A Chemo-Enzymatic Synthesis of β-D-Arabinofuranosyl Purine Nucleosides. *Synthesis* **2011**, (10), 1555-1560; doi: 10.1055/s-0030-1260010.
5. Averett, D.R.. Koszalka, G.W., Fyfe, J.A.. Roberts, G.B., Purifoy, D.J.M. and Krenitsky, T.A. 6-Methoxypurinc arabinoside as a selective and potent inhibitor of varicella-zostervirus. *Antimicrob Agents Chemother*. **1991**, *35* (5), 851–857. doi: 10.1128/AAC.35.5.851.
6. Averett, D.R.; Jones, L.A.; Moorman, A. R.; Koszalka, G.W.; Krenitsky, Th.A. Antiviral compounds. EP 0 294 114 B1. 11.09.1996 Bulletin 1996/37; THE WELLCOME FOUNDATION Ltd. London NW1 2BP (GB); Priority: 30.05.1987 GB 8712745.
